# Validating subject-specific knee models from *in vivo* measurements

**DOI:** 10.3389/fbioe.2025.1554836

**Published:** 2025-08-14

**Authors:** Thor E. Andreassen, Donald R. Hume, Landon D. Hamilton, Stormy L. Hegg, Sean E. Higinbotham, Kevin B. Shelburne

**Affiliations:** ^1^ Center for Orthopaedic Biomechanics, Department of Mechanical and Materials Engineering, University of Denver, Denver, CO, United States; ^2^ Assistive and Restorative Technology Laboratory, Department of Physical Medicine and Rehabilitation, Mayo Clinic, Rochester, MN, United States

**Keywords:** finite element model, validation, subject-specific, knee, computational modeling and simulation, *in vivo*, optimization, digital twin

## Abstract

Despite the documented consequences of modeling decisions on the performance of computational models in orthopaedics and biomechanics, the influence of the input data has largely been ignored. Modeling the living knee is limited by methods to measure *in vivo* the quantities needed for ligament calibration; yet, this may be possible with new devices focused on non-invasive measurement of knee laxity. These devices offer measurements similar to those commonly obtained from cadaveric specimens but are limited by what can be practically and safely obtained from a living subject. Validation of models calibrated with *in vivo* data is crucial and increasingly important as personalized modeling becomes the basis for proposed digital twins, and *in silico* clinical trial workflows. To support our overall goal of building subject-specific models of the living knee, we aimed to show that subject-specific computational models calibrated using *in vivo* measurements would have accuracy comparable to models calibrated using *in vitro* measurements. Two cadaveric knee specimens were imaged using a combination of computed tomography (CT) and surface scans. Knee laxity measurements were made with a custom apparatus used for the living knee and from a robotic knee simulator. Models of the knees were built following previous methods and then calibrated with either laxity data from the *in vitro* robotic knee simulator (RKS) or from the *in vivo* knee laxity apparatus (KLA). Model performance was compared by simulation of various activities and found to be similar between models calibrated with laxity targets from the RKS and the KLA. Model predictions during simulated anterior-posterior laxity tests differed by less than 2.5 mm and within 2.6° and 2.8 mm during a simulated pivot shift. Still, differences in the predicted ligament loads and calibrated material properties emerged, highlighting a need for methods to include ligament load as part of the calibration process. Overall, the results showed that currently available methods of measuring knee laxity *in vivo* are sufficient to calibrate models comparable with existing *in vitro* techniques, and the workflows described here may provide a basis for modeling the living knee. The experimental data, models, results, and tools are publicly available.

## Graphical Abstract

A graphical abstract outlining the key points of the work
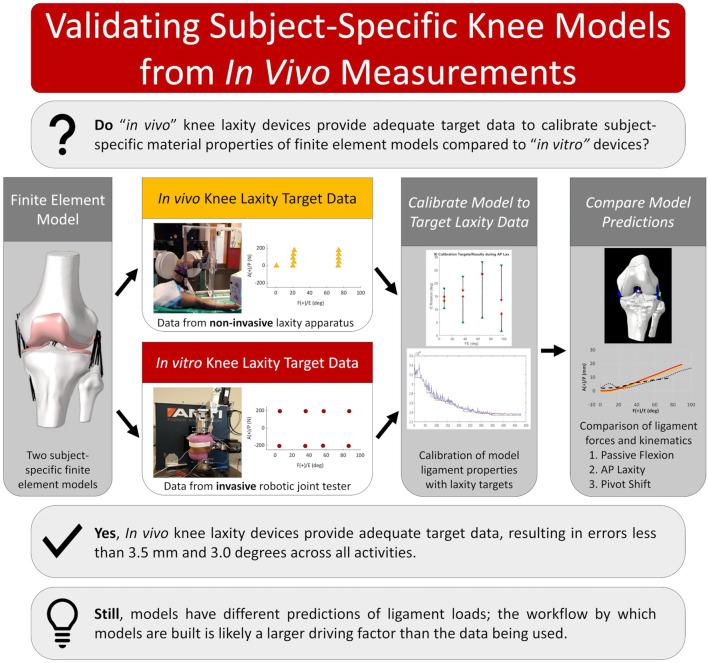

## 1 Introduction

There is a widespread endeavor to create personalized models that mimic individuals with clinically meaningful accuracy. These efforts seek to more accurately represent the individual variability affecting functional outcomes to ultimately improve personalized medicine. Model personalization hinges on uniquely recreating an individual by obtaining subject-specific measurements and using those to recreate an individual’s geometry, material properties, and loading and boundary conditions to develop digital twins (Hassani et al., 2022; Sun et al., 2022; Viceconti et al., 2024). Many different modeling workflows with differing levels of personalization have been utilized to model human biomechanics motivating investigations into their reproducibility, validity, accuracy, and limitations before their adoption in clinical settings (Anderson et al., 2007). In knee biomechanics, personalized models of the living knee are most often constructed from medical imaging, such as computed tomography (CT), magnetic resonance imaging (MRI), or statistical tools (Van Oevelen et al., 2023). Modeled knee structure material properties are then calibrated by matching predictions of joint level dynamic behaviors with those experimentally measured by adjusting parameters in constitutive models (Erdemir et al., 2019). Researchers have aimed to understand the effect of different model parameters (Farshidfar et al., 2022) on model dynamic behavior with investigations into ligament representation and material properties (Naghibi Beidokhti et al., 2017; Peters et al., 2018), bone material properties (Peters et al., 2018; Kluess et al., 2019), cartilage representation and material properties (Klets et al., 2016; Peters et al., 2018) and the choice of constitutive models and representations for other structures such as the meniscus (Elmukashfi et al., 2022). However, the influence of the experimental data used for calibration on the predictive abilities of models has largely been ignored. Specifically, calibration of the ligament material properties is necessary to improve model predictions of individual kinematics and tissue loads, and absence of personalized properties may result in poor model predictions (Gardiner and Weiss, 2003; Andreassen et al., 2023).

Calibration of ligament material properties to recreate the kinetic force-displacement behavior of the knee requires obtaining the necessary measurements. Likely for this reason, models of the living knee frequently use ligament material properties from the literature without direct calibration (mathematical optimization of ligament material properties) to the individuals of interest (Carey et al., 2014; Kang et al., 2017; Shu et al., 2018; Esrafilian et al., 2020; Theilen et al., 2023). Notably, Ali et al. calibrated ligament material properties to match the kinematics of passive knee flexion measured from their modeled subjects (Ali et al., 2020). Nevertheless, model predictions of loaded kinematics were not directly compared against physical measurements and instead relied on predictions from musculoskeletal modeling. Alternatively, ligament material properties in specimen-specific models of the knee are calibrated from measurements readily obtained from cadaveric tissue (Bloemker et al., 2015; Harris et al., 2016; Kia et al., 2016; Kluess et al., 2019; Razu et al., 2023). Various specimen-specific models of cadaver knees have been calibrated utilizing data from ligament forces (Kia et al., 2016; Razu et al., 2023), zero-load ligament lengths (Bloemker et al., 2015), joint distraction of the bones (Zaylor et al., 2019), or large numbers of trials of high-accuracy force-displacement measurements from robotic knee simulators (Harris et al., 2016; Chokhandre et al., 2022; Andreassen et al., 2023), all of which are impractical methodologies in living people. Methods to calibrate models of the living knee using measurements available *in vivo* have not been validated against measurements available *in vitro*.

Modeling the living knee is confined by the limited means to measure *in vivo* the quantities needed for ligament calibration. Fortunately, subject-specific calibration of knee models may be possible with recent improvements in non-invasive measurement of knee laxity *in vivo* from the creation of several new devices (Kupper et al., 2016; Moewis et al., 2016; Pedersen et al., 2019; Andreassen et al., 2021; Shamritsky et al., 2023; Imhauser et al., 2024). These devices, and others, offer significant improvements over previous laxity measurement devices, such as the KT-1000 (Collette et al., 2012) and offer measurements similar to those obtained from cadaveric specimens. However, compared with existing cadaveric laxity measurement methods, the measurements from *in vivo* devices are limited in the number of samples, joint angles, and loading conditions that can be practically and safely obtained from a living subject. Validation (comparison of model predictions against experimental data or previously validated methods) of models calibrated with *in vivo* data by comparison to models calibrated with *in vitro* datasets is crucial and increasingly important as personalized modeling becomes the basis for proposed digital twins, and *in silico* clinical trial workflows.

To support the long-term goal of creating personalized models of the living knee, this study evaluated whether models with ligaments calibrated to laxity measurements obtained with *in vivo* methods are comparable to models calibrated from laxity measurements obtained with conventional *in vitro* methods. This was accomplished in three steps. First, subject-specific finite element models of two cadaveric specimens were developed using a combination of imaging data and methods matching that of previous knee modeling work. Second, and the key piece of this work, these models were calibrated to *in vitro* laxity measurements collected with a robotic knee simulator, and then separately calibrated to laxity measurements collected with an *in vivo* device. Lastly, model predictions were compared between the two calibration scenarios for similarity and accuracy of kinematics and ligament forces during passive knee flexion, anterior-posterior laxity, and a pivot shift test. We hypothesized that methods for *in vivo* measurement of knee laxity would allow calibration that produces accuracy comparable with calibration from *in vitro* measurements. The experimental data, working models, results, and tools are publicly available to encourage model reproducibility.

## 2 Materials and methods

### 2.1 Overview

To evaluate the use of *in vivo* laxity measurements for model creation and calibration, finite element models (FEM) were compared between two calibration methodologies: 1) FEM calibrated to knee laxity measurements obtained using *in vivo* methods; 2) FEM calibrated to knee laxity measurements obtained from a robotic knee simulator. Geometries of two cadaveric knee specimens (Table 1) were obtained from a combination of lower-extremity CT scans, and surface scans of the bones and soft tissues. Measurements for calibration were obtained using a previously validated knee laxity apparatus (KLA) (Andreassen et al., 2021) designed to measure knee laxity *in vivo* and a robotic knee joint simulator (RKS). *In vivo* laxity experimentation was recorded first using two intact lower body cadaveric specimens using the KLA, and then subsequent dissection performed to facilitate RKS testing on the same specimens.

**TABLE 1:** Donor specifics for knee models. Specimen IDs are used for Supplementary Material, including model files, where the data are referred to using the Specimen ID rather than Specimen 1 and Specimen 2.

	Specimen 1	Specimen 2
Specimen ID	S192803	S193761
Modeled side	L	L
Sex	M	M
Age (years)	29	64
Height (cm)	188	178
Weight (kg)	113.4	56.2
BMI (kg/m^2^)	32.1	17.8

**FIGURE 1:**
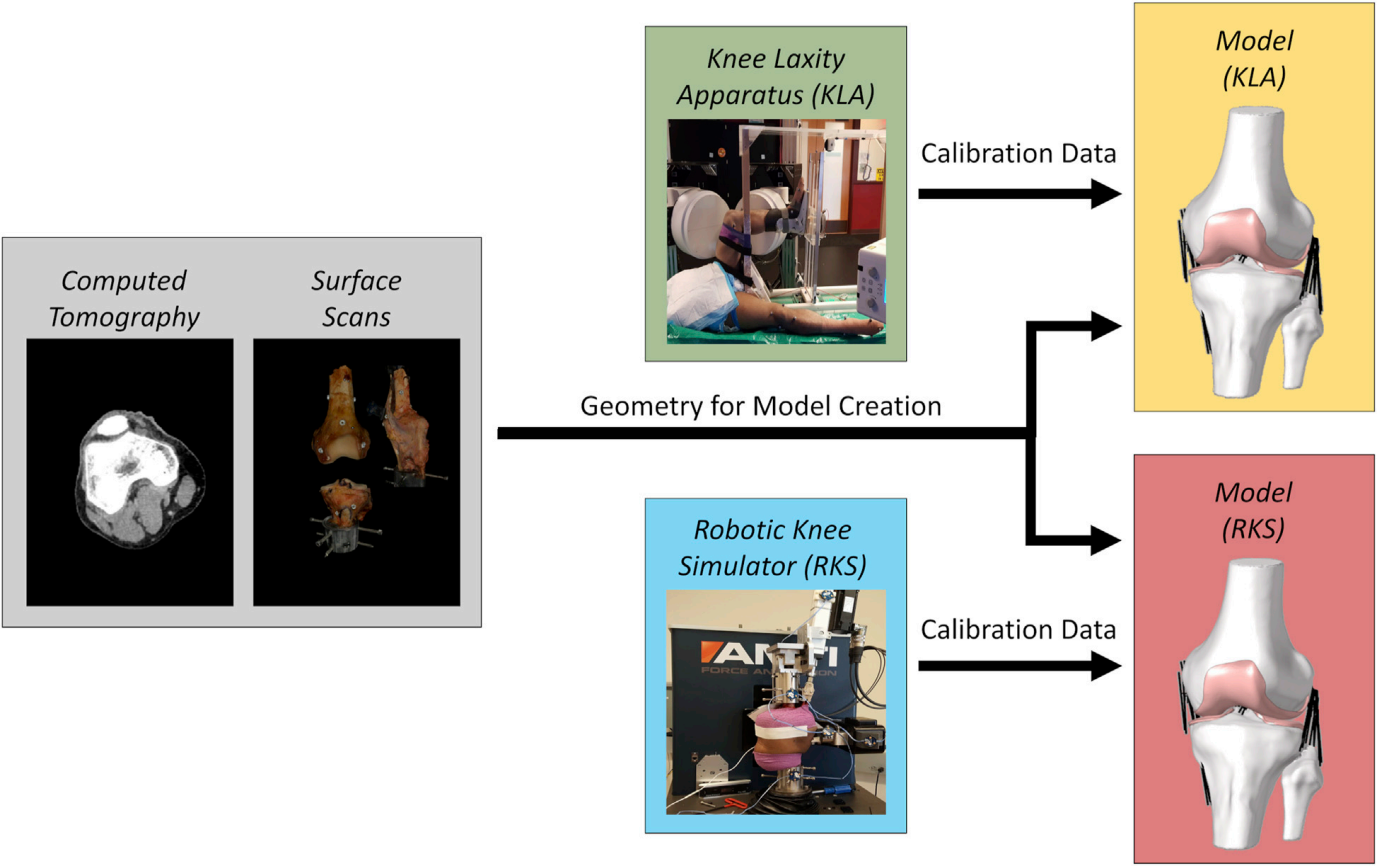
Modeling workflow for each knee specimen. Gray regions are the original imaging data used to create models of the two knee specimens. The combined CT scans and surface scans were used to create model geometries for both specimens. Green and blue regions are the source of experimental laxity measurements used for calibration, namely a knee laxity apparatus (KLA) and a robotic knee simulator (RKS). Yellow and red regions are the resulting models defined using the corresponding imaging data and the different knee laxity data sources.

The CT and surface scans were used to create model geometries for both specimens. These models were then calibrated against two different laxity datasets. White light surface scanning was chosen as a rigorous means of producing the best possible geometry to isolate the impact of force-displacement calibration from the notable variability inherent in geometric reconstruction from *in vivo* sources (Rooks et al., 2021) and its influence on calibration (Andreassen et al., 2023). In one case, models were calibrated to laxity measurements from the knee laxity apparatus, known as the “KLA” models. The other case was calibrated to laxity measurements from the robotic knee simulator, known as the “RKS” models (Figure 1). The two models were then used to predict anterior-posterior laxity at various knee flexion angles and a passive knee flexion. Kinematics and ligament force predictions were compared. Additionally, models were used to predict a simulated pivot shift and resulting kinematics and ligament loads compared.

The knee modeling process followed the Team DU workflow from the KneeHub project (SimTK: Reproducibility in Simulation-Based Prediction of Natural Knee Mechanics: Project SimTK, 2018; Erdemir et al., 2019; Rooks et al., 2021; Andreassen et al., 2023) to allow for a comparison with previous work that examined the differences in modeling strategy. The following sections describe the experimental and modeling workflow.

### 2.2 Experimental data collection

The experimental data were collected as part of previous work (Andreassen et al., 2021) and is summarized herein. Two non-frozen male pelvis-to-toes cadavers (Table 1) were obtained with no history of musculoskeletal ailments. Prior to testing, specimens underwent CT scans (Figure 1). CT scans were collected axially (Siemens SOMATOM Perspective, Erlangen, Germany) with approximately 0.75 mm × 0.75 mm in-plane resolution and a 0.6 mm axial resolution from approximately L5 to the toes of both legs. Bone geometries were segmented from CT scans using a combination of global thresholding and manual segmentation methods and exported as STLs (Simpleware ScanIP, Synopsys, Sunnyvale, CA).

Whole limb specimens were placed in a custom knee laxity apparatus (KLA) (Andreassen et al., 2021) designed to measure laxity in the living knee noninvasively. To simulate a standard knee laxity data collection with a living subject recreating *in vivo* conditions as much as possible, a series of loads was applied to the anterior, internal, and external degrees of freedom (DOF) of the tibia at 30 and 90° of knee flexion. Maximum loads were approximately 175 N for anterior and 5.5 N*m for internal and external as measured via load cell. To approximate a passive knee extension while stereo radiography images were recorded, a cuff was placed around the ankle and attached with a cable and rod to manually push the knee to deep flexion (∼150°) and pull to full extension. This simulated passive knee extension is later referred to as the “*Intact Leg Experimental*” kinematics. The resulting displacements for all DOF were recorded using 3D image tracking techniques from high-speed stereo radiography (HSSR) images (Ivester et al., 2015; Kefala et al., 2017).

Immediately following these measurements, specimens were dissected, leaving approximately 230 mm of soft tissue and bone intact above the knee joint line and 200 mm below the knee joint line. Each knee specimen was cemented into custom femur and tibia-fibula fixtures and affixed to a VIVO robotic knee simulator (RKS) (AMTI, Watertown, MA). Additionally, a custom quadriceps actuator (Behnam et al., 2024) was affixed to the quadriceps tendon to simulate the passive tension in the quadriceps tendon (McKay et al., 2010). The joint simulator applied laxity loads for anterior-posterior (AP), internal-external (IE), and varus-valgus (VrVl) between 0–120° of knee flexion in 15-degree increments. Maximum loads applied were approximately 200 N for AP (Mouton et al., 2015), 7.5 N*m for IE, (Wang et al., 2020), and 10 N*m for VrVl (Schmitz et al., 2008) and measured using a built-in 6 DOF load cell. The resulting displacements for all DOF were recorded using an Optotrak motion capture system (NDI, Ontario, Canada). Following laxity testing, the passive range of motion of the knee was recorded in the simulator with no loads applied and is later referred to as the “*Dissected Knee Experimental*” kinematics.

The laxity values used during the model calibration (described below) were selected as a subset of the overall measurements collected from experimentation and differed between the RKS and KLA data sources because of experimental constraints and the model optimization procedures. From the KLA data, laxity values used for model calibration were the anterior and IE knee laxity at 30 and 90° of knee flexion at various load levels. From the RKS data, laxity values used for model calibration were the AP, IE, and VrVl laxity at 0, 30, 60, and 90° of knee flexion at the maximum and minimum loads measured. Due to experimental limitations, the target used was chosen at 75 instead of 90° of knee flexion in some cases. In all models, an additional calibration target was placed at 0° of knee flexion taken from the kinematics of the passive range of motion at full extension.

**FIGURE 2:**
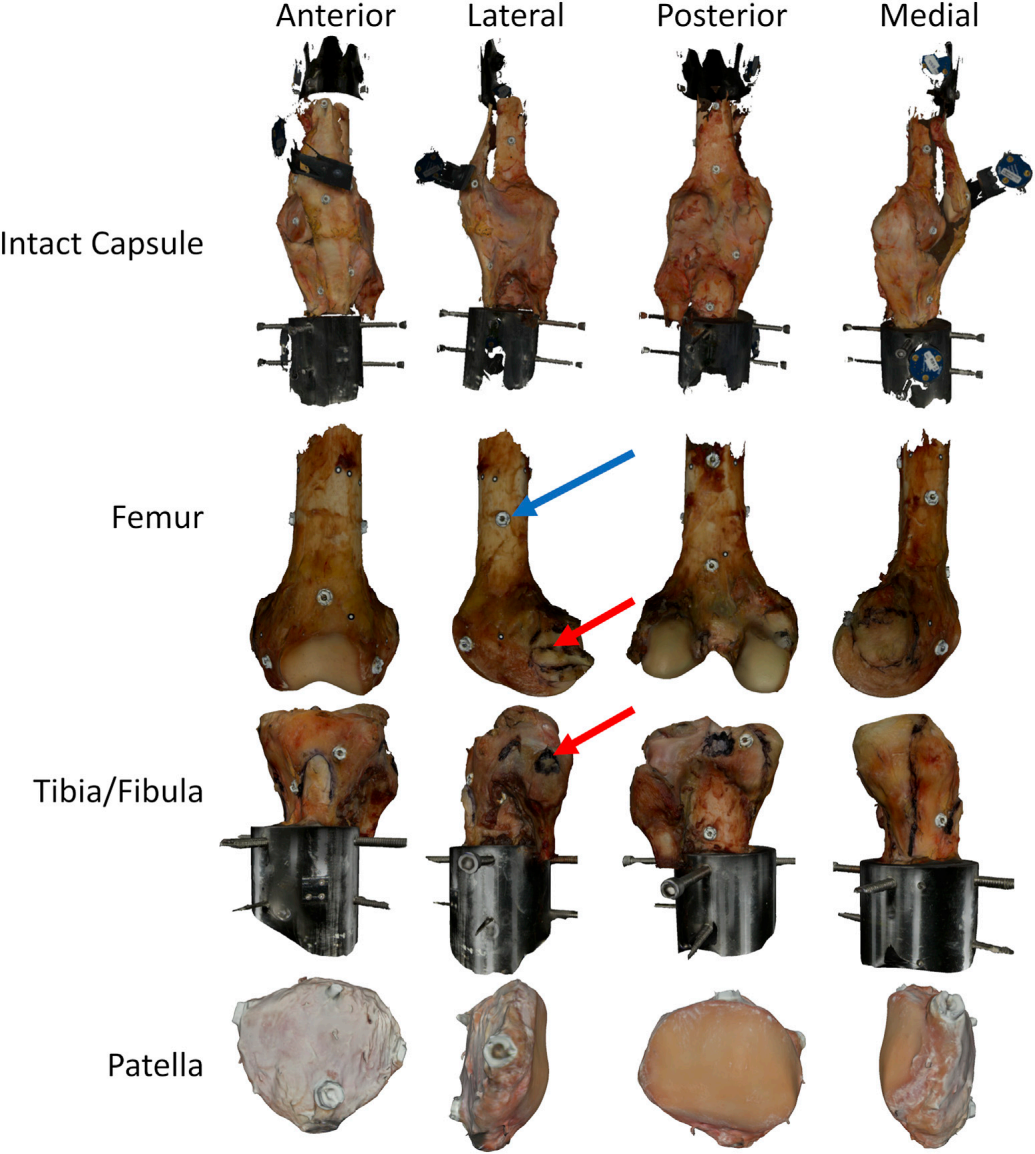
Surface scans of a knee at various stages of dissection, intact with capsule, femur only, tibia/fibula only, and patella only. The black highlighted regions on the bones represent different ligament attachment sites identified during the dissection and marked on the specimens using a permanent marker. Bones include fiducial screws and dots to allow for improved registration after the fact, and the combining of the original surface data collected from the scanner. The red arrows highlight one of the attachment sites (LCL) identified during dissection. The blue arrow highlights one of the fiducial screws used for registration. In all cases, the fiducial screws shown were added after testing to allow for superior registration. As such, they did not impact the joint laxity measurements.

The knee specimens were further dissected after experimentation, leaving the bones, ligaments, and knee capsule intact. A white-light scanner (Artec Space Spider, Artec, Santa Clara, CA) was used to scan the surface of the knees (Figure 2). Then, the soft-tissue structures were removed, and ligament attachment sites of the major knee ligaments and tendons (patellar tendon) were outlined on the bones, and the bones were scanned again (Figure 2). Fiducial screws and stickers were added to the bones prior to scanning to assist registration, but did not affect the experimental kinematics measurements. The scanner provided high-resolution color texture surfaces of the bone and the exact locations of soft-tissue attachment outlines on the bone (Figure 2), with reconstruction accuracy comparable to micro-CT (Hayes et al., 2016). Scans of the full intact knee capsule as well as separate scans for the femur, tibia-fibula, and patella were all collected for both specimens (Figure 2). The resulting geometries were exported as STLs.

**FIGURE 3:**
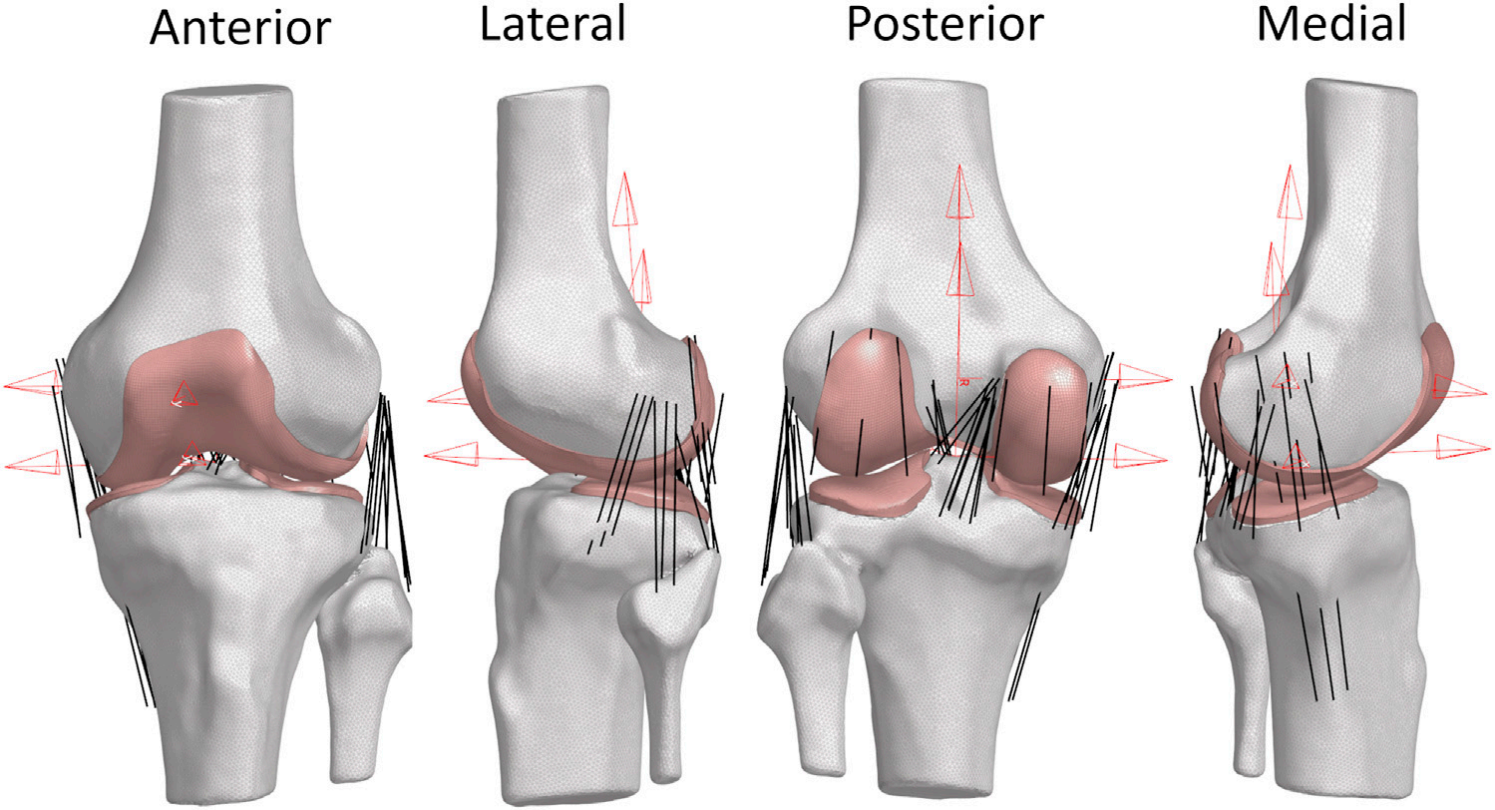
Views of Specimen 1 FEM. (Red) Femoral and tibial coordinate system definitions using transepicondylar axis (TEA) for the femur coordinate system and the standard Grood and Suntay coordinate system for the tibia. These coordinate systems were defined using the full-length bones. (White) Bone meshes as 2D triangular surface elements. (Pink) Cartilage meshes as 3D hexahedral volume elements. (Black) Ligaments as 1D non-linear tension-only connector elements.

Geometries from the CT were used to create local bone coordinate systems in the transepicondylar (TEA) axis coordinate system (Figure 3) following the joint coordinate system convention from Grood and Suntay (Grood and Suntay, 1983). All kinematics from the KLA and the RKS testing were represented in the same local coordinate system of the bones.

### 2.3 Geometry identification

Geometries of the bones were created from a combination of CT and surface scans. Geometries of the bones from the surface scan were first aligned to the position of the bones in the CT with a combination of manual and automatic registration using an iterative closest point (ICP) algorithm in MATLAB (Mathworks, Natick, MA). Final model geometries of the bones were created by cropping the bones obtained from the CT images to the region around the knee (approximately 150 mm above and below the joint line). Geometries of cartilage were obtained by Boolean subtraction of the aligned CT of the bones with the corresponding surface scans of the bones and cartilage (Kia et al., 2016). In all cases, geometries were smoothed and fixed (removal of poor-quality elements, removal of inaccuracies from segmentations, etc.) using a combination of MeshMixer (Autodesk, San Francisco, CA) and MeshLab (Cignoni et al., 2008). Marked attachment site regions from the surface scans for the major ligaments (ACL, LCL, MCL, PCL) and patellar tendon were projected along surface normal directions to the CT bones to determine the approximate attachment on the true boney surface. For ligaments that could not be easily identified during experimentation, approximate attachment sites were identified using descriptions from the literature, summarized in Table 2 (LaPrade et al., 2003; 2007; 2021; De Maeseneer et al., 2004; Petersen and Zantop, 2007; Liu et al., 2010; Claes et al., 2013; Chahla et al., 2020). In total, the model contains the following 14 ligaments: anteromedial bundle of anterior cruciate ligament (ACL_AM), posterolateral bundle of anterior cruciate ligament (ACL_PL), lateral collateral ligament (LCL), superficial anterior fiber of medial collateral ligament (sMCL_A), superficial middle fiber of medial collateral ligament (sMCL_M), superficial posterior fiber of medial collateral ligament (sMCL_P), deep bundle of medial collateral ligament (dMCL), anterolateral bundle of posterior cruciate ligament (PCL_AL), posteromedial bundle of posterior cruciate ligament (PCL_PM), anterolateral structure (ALS), popliteofibular ligament (PFL), posterior oblique ligament (POL), medial posterior capsule (PCAP_M), and the lateral posterior capsule (PCAP_L).

**TABLE 2:** Modeled knee ligaments and anatomical descriptions of attachment sites and any adjustments used.

Ligament	Ligament major group	Ligament abbreviation	Literature description of ligament origin	Literature description of ligament insertion	Adjustments to attachment sites
Anteromedial Bundle of ACL	ACL	ACL_AM	Posterior portion of the lateral femoral condyle. Posterior to lateral intercondylar ridge. Superior to bifurcate ridge (Petersen and Zantop, 2007)	Centralized in the ML direction on the tibial plateau, at approximately 30% of the total AP length of the tibia from the anterior side (Petersen and Zantop, 2007)	None
Posterolateral Bundle of ACL	ACL	ACL_PL	Posterior portion of the lateral femoral condyle. Posterior to lateral intercondylar ridge. Inferior to bifurcate ridge (Petersen and Zantop, 2007)	Centralized in the ML direction on the tibial plateau, at approximately 44% of the total AP length of the tibia from the anterior side (Petersen and Zantop, 2007)	None
Main Bundle of LCL	LCL	LCL	Approximately 1.4 mm superior and 3.1 mm posterior to the lateral epicondyle of the femur (LaPrade et al., 2003)	Inserts into fibula head approximately 8 mm posterior of the anterior portion of the fibular head, and approximately 28 mm distal to the fibular head apex (LaPrade et al., 2003)	None
Superficial Anterior Fiber of MCL	MCL	sMCL_A	Slightly superior and anterior to the medial epicondyle of the femur (Liu et al., 2010)	Anterior region of the medial side of the tibia approximately 6 cm distal to the tibial joint line. Additional insertion around the most medial portion of the tibial plateau (Liu et al., 2010)	In cases with a rapidly narrowing tibia, insertion was chosen to be the proximal attachment of the superficial MCL, rather than the distal one to approximate correct line of action
Superficial Middle Fiber of MCL	MCL	sMCL_M	Slightly superior to the medial epicondyle of the femur (Liu et al., 2010)	Middle region of the medial side of the tibia approximately 6 cm distal to the tibial joint line. Additional insertion around the most medial portion of the tibial plateau (Liu et al., 2010)	In cases with a rapidly narrowing tibia, insertion was chosen to be the proximal attachment of the superficial MCL, rather than the distal one to approximate correct line of action
Superficial Posterior Fiber of MCL	MCL	sMCL_P	Slightly superior and posterior to the medial epicondyle of the femur (Liu et al., 2010)	Posterior region of the medial side of the tibia approximately 6 cm distal to the tibial joint line. Additional insertion around the most medial portion of the tibial plateau (Liu et al., 2010)	In cases with a rapidly narrowing tibia, insertion was chosen to be the proximal attachment of the superficial MCL, rather than the distal one to approximate correct line of action
Deep Bundle Fiber of MCL	MCL	dMCL	Posterior and inferior to the superficial MCL and the medial epicondyle (Liu et al., 2010)	Medial aspect of the tibial plateau approximately 6 mm from the tibial joint line (Liu et al., 2010)	None
Anterolateral Bundle of PCL	PCL	PCL_AL	Located on the anterior side of the medial condyle in the intercondylar fossa. Inferior to the medial intercondylar ridge. Anterior to the medial arch point (Chahla et al., 2020; LaPrade et al., 2021)	Anterolateral to PCL_PM and near the posterior medial edge of the lateral meniscus (Chahla et al., 2020; LaPrade et al., 2021)	None
Posteromedial Bundle of PCL	PCL	PCL_PM	Located on the anterior side of the medial condyle in the intercondylar fossa. Inferior to the medial intercondylar ridge. Posterior to the medial arch point (Chahla et al., 2020; LaPrade et al., 2021)	Edge of the champagne glass dropoff (CGD) of the tibial plateau in the intercondylar facet (Chahla et al., 2020; LaPrade et al., 2021)	None
Anterolateral Structure	ALS	ALS	Originate on the lateral epicondyle of the femur just anteriorly to the origin of the LCL. In many cases, the origin is more superior and joins with the portion of the LCL (Claes et al., 2013)	Posterior to Gerdy’s tubercle on the tibial plateau. Approximately found at the intersection of a ray cast between the Gerdy’s Tubercle and the fibular head (Claes et al., 2013)	None
Popliteofibular Ligament	PFL	PFL	Approximately 18.5 mm from the LCL origin on the femur in the inferior/anterior direction (LaPrade et al., 2003)	Inserts into the fibula approximately 3 mm inferior to the apex of the fibula head on the anteromedial slope (LaPrade et al., 2003)	PFL femoral attachment was moved approximately to the medial epicondyle to approximate the line of action of the force of the combined popliteofibular ligament and popliteal tendon rather than true anatomic accuracy
Posterior Oblique Ligament	POL	POL	Superior and posterior to the origin of the Superficial MCL. Approximately 8 mm inferior and 6.4 mm posterior to the adductor tubercle and 1 mm inferior and 3 mm anterior to the gastrocnemius tubercle (LaPrade et al., 2007)	Slightly inferior to the tibial posteromedial portion of the tibial plateau near the posteromedial portion of the medial meniscus (LaPrade et al., 2007)	None
Medial Posterior Capsule	PCAP	PCAP_M	Originates on the posteromedial portion of the femoral cortex a few centimeters above the most superior portion of the femoral cartilage (De Maeseneer et al., 2004)	Posteromedial portion of the tibial plateau, approximately 1–2 cm below the knee joint line (De Maeseneer et al., 2004)	Femoral origin was moved to the edge of the posterior condyles of the femur, where the contact of the capsule with the condyles would occur
Lateral Posterior Capsule	PCAP	PCAP_L	Originates on the posterolateral portion of the femoral cortex a few centimeters above the most superior portion of the femoral cartilage (De Maeseneer et al., 2004)	Posterolateral portion of the tibial plateau, approximately 1–2 cm below the knee joint line	Femoral origin was moved to the edge of the posterior condyles of the femur, where the contact of the capsule with the condyles would occur

### 2.4 Finite element model development

#### 2.4.1 Overview

**FIGURE 4:**
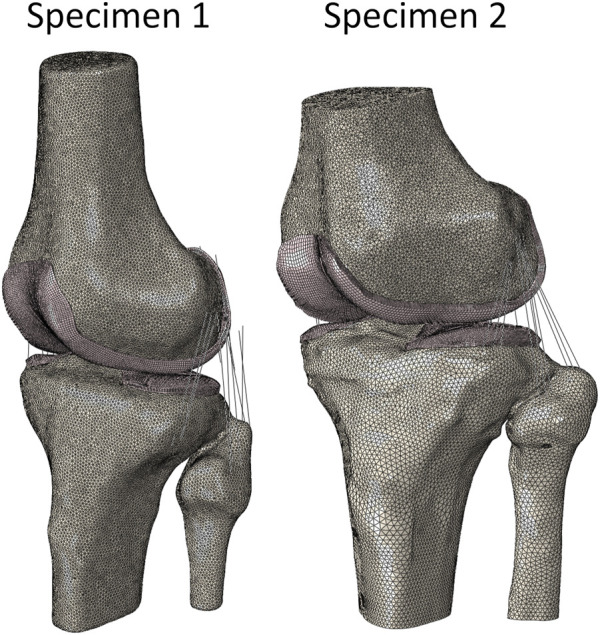
FEM of Specimen 1 and Specimen 2. (White) 2D bone elements (Pink) 3D cartilage elements (Black) 1D ligament elements.

FEM of the knee was created in Abaqus Explicit (Dassault Systemes, France) using a previously described modeling workflow (Rooks et al., 2021) (Figures 3, 4). Models of each specimen were created in the initial position of the bones defined by their full-extension position in the CT. Rigid body reference nodes were defined for the femur and tibia/fibula geometries. Cylindrical joints were created between the femur and tibia/fibula rigid body nodes following the Denavit-Hartenberg convention described by Grood and Suntay (GS) (Grood and Suntay, 1983). These joints allowed the application of loads or displacements to each DOF along the cylindrical joints for medial-lateral (ML), anterior-posterior (AP), and superior-inferior (SI) or as torques or rotations around the cylindrical joints for flexion-extension (FE), varus-valgus (VrVl), and internal-external (IE) (Andreassen et al., 2023). The bony surface of the cartilage geometry was rigidly fixed to the rigid body nodes for the femoral and tibial cartilage. Ligaments were defined with 1D connectors and rigidly fixed to the bones. Contact was modeled between the cartilage surfaces of the tibia and the femur with a friction coefficient of 0.03 (Basalo et al., 2006).

#### 2.4.2 Bone meshes

Bones of the femur and a combined tibia and fibula were modeled as rigid triangular surfaces (R3D3 elements in Abaqus) rigidly fixed to a rigid body node following previous work (Harris et al., 2016; Rooks et al., 2021). Elements of geometries were approximately 1.5 mm in size.

#### 2.4.3 Cartilage meshes

Cartilage geometries generated for the distal femur and the medial and lateral proximal tibia were exported to Hypermesh (Altair, Troy, MI), and the articular surface and bony surfaces were identified. A quadrilateral mesh was created on the bony surface. The quadrilateral mesh and the original articular and bony surfaces were used in Hypermesh to create 3D reduced-integration hexahedral cartilage meshes (C3D8R elements in Abaqus). While stress was not critical to this study, the cartilage was meshed as hexahedral elements with appropriate element sizes to allow for future use for stress analysis with non-linear material models. Following previous methods (Halloran et al., 2005; Fitzpatrick et al., 2010; Huff et al., 2020), the cartilage was modeled with a calibrated (unique to each specimen) tri-linear pressure overclosure material property to improve computational performance. The details of this calibration are provided in the Supplementary Material.

A mesh convergence study was performed following the recommendations for calculation verification from Anderson et al. (2007). Convergence of contact area, contact pressure, total displacement, and von Mises stress was verified, and results are reported in the Supplementary Material. All cartilage geometries had target element lengths at the largest between 1 mm and 0.5 mm.

After the hexahedral meshes were created from the original triangulated surfaces, initial overclosures were observed between the femoral and tibial cartilage. Overclosures often create instability and convergence problems for explicit finite element analysis (FEA). Our previously developed and publicly available code package and corresponding algorithm using generalized regression neural networks (GRNNs) was used to remove initial overclosures between cartilage geometries via equal weighting of the resulting deformations between tibial and femoral cartilage (Andreassen et al., 2024b).

#### 2.4.4 Ligament connectors

Ligaments were modeled as non-linear 1D tension-only spring connectors (Axial type CONN3D2 elements in Abaqus) like those previously used (Blankevoort and Huiskes, 1996) and formally described by Yu et al. (2001). Ligaments were modeled with a reference strain parameter defining the initial tension present in the ligament in its initial configuration and the stiffness of the ligament in the linear region (Table 3). The range of values used for the reference strain and ligament stiffness was approximately the same as those reported in the “Knee Model Calibration Specification” document for Team DU in the KneeHub Project (SimTK: Reproducibility in Simulation-Based Prediction of Natural Knee Mechanics: Project SimTK, 2018; Erdemir et al., 2019; Rooks et al., 2021; Andreassen et al., 2023). A constant quadratic toe-in region was created for all ligaments with an assumed strain parameter of 0.03 (Blankevoort et al., 1991). Ligaments were separated into models of individual bundles based on anatomical descriptions, with several fibers modeled for each bundle. The location of the individual fibers was determined by visually identifying the approximate major axis of the ligament attachment region and choosing points equidistant along the major axis based on the number of desired fibers to approximate the span of the overall region (Supplementary Material). Reference strain and stiffness parameters were unique for each ligament, but all fibers within a single bundle shared the same material properties. Ligament attachments were tied to the respective bone’s rigid body nodes (multi-point constraint beam type in Abaqus).

**TABLE 3:** Modeled ligaments organized by bundle, number of modeled fibers, and design variable assigned to ligament material parameter. X1 represents the first design variable, X2 represents the second design variable, and so on.

Ligament	Ligament major group	Ligament abbreviation	Number of fibers	Reference strain design variable	Reference strain range	Stiffness design variable	Stiffness range (N/mm)
Anteromedial Bundle of ACL	ACL	ACL_AM	2	X1	[0.95–1.25]	X15	[50–150]
Posterolateral Bundle of ACL	ACL	ACL_PL	2	X2	[0.95–1.25]	X16	[50–150]
Main Bundle of LCL	LCL	LCL	3	X3	[0.55–1.15]	X17	[60–200]
Superficial Anterior Fiber of MCL	sMCL	sMCL_A	1	X4	[0.70–1.05]	X18	[40–180]
Superficial Middle Fiber of MCL	sMCL	sMCL_M	1	X5	[0.70–1.05]	X18	[40–180]
Superficial Posterior Fiber of MCL	sMCL	sMCL_P	1	X6	[0.70–1.05]	X18	[40–180]
Deep Bundle Fiber of MCL	dMCL	dMCL	3	X7	[0.55–1.05]	X19	[40–180]
Anterolateral Bundle of PCL	PCL	PCL_AL	2	X8	[0.85–1.15]	X20	[30–100]
Posteromedial Bundle of PCL	PCL	PCL_PM	2	X9	[0.85–1.25]	X21	[30–100]
Anterolateral Structure	ALS	ALS	2	X10	[0.75–1.25]	X22	[20–125]
Popliteofibular Ligament	PFL	PFL	3	X11	[0.85–1.15]	X23	[10–90]
Posterior Oblique Ligament	POL	POL	2	X12	[0.75–1.15]	X24	[30–95]
Medial Posterior Capsule	PCAP	PCAP_M	3	X13	[0.85–1.25]	X25	[50–100]
Lateral Posterior Capsule	PCAP	PCAP_L	3	X14	[0.85–1.25]	X26	[50–100]

#### 2.4.5 Simulation of knee motion

The position of the tibia and fibula rigid body nodes was rigidly fixed in all DOF (boundary encastre in Abaqus) during every step, while the position of the femur was determined by a series of three cylindrical (rotation and translation about a single axis) joint connectors (Cylindrical type CONN3D2 elements in Abaqus). All DOF for the joint connectors were placed in load control except for the FE connector, which applied the desired knee flexion angle in displacement control. All simulations of knee dynamics utilized two sequential steps in Abaqus Explicit.

The first step, a settling step, placed the model in equilibrium and resolved any initial contact penetration before simulation of a target load and pose in step two. The first step began with the bones in their initial CT full-extension pose, and applied a desired compression level in the SI direction to the cylindrical SI connector (connector load in Abaqus) using a load starting at 0 N and linearly ramping to the desired compression level causing the femur to compress into the tibia. The model was highly damped in the first step to reduce vibrations caused by ligament tension as the model settled into a stable initial pose. Damping was applied to the joint connectors (connector damping in Abaqus) for the translation and rotational DOF. To ensure the high damping did not affect the motion in the second step, the damping was defined as dependent on temperature. A high temperature during the first step resulted in significant damping (100 N*s/mm for translation and 100 N*s*mm/rad for rotation).

During the second step, the knee was flexed to the desired knee angle (based on the angle for the specific target described in more detail below) by applying a rotation about the cylindrical FE connector in displacement control (connector motion in Abaqus) causing the rigid body node of the femur to rotate, as well as all tied ligaments. Target loads from the corresponding model target (described in detail below) were applied to the respective DOF in force control (connector load and CLOAD in Abaqus). This step had a low temperature resulting in negligible damping. The load in each DOF was linearly increased to the target value and held constant for the final 30% of the step.

### 2.5 Model Calibration

**FIGURE 5:**
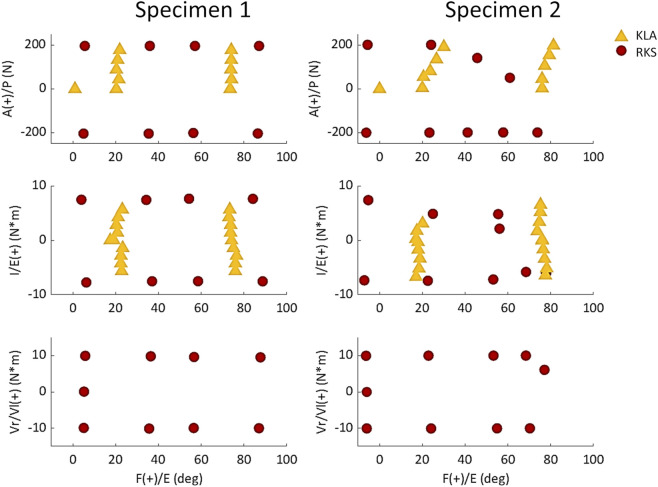
Chosen laxity targets for knee calibration selected from measurements made with the KLA and RKS. Points are the loads and corresponding knee angles of the true experimental data used as targets for the model calibration instead of the approximate knee flexion angle and loads.

Ligament reference strain and stiffness parameters were calibrated in an optimization process that simulated knee model movement in response to target loading conditions and minimized the error between the measured and predicted kinematics (Andreassen et al., 2023). Specifically, a set of calibration targets (matched kinematics and load data) were defined from the laxity measurements described above at discrete knee flexion angles and levels of applied load. Separate calibration targets were created from the laxity measurements made using the KLA and the RKS (Figure 5).

The optimization process was managed in MATLAB. For each iteration of the optimization process, a custom MATLAB script was used to set the ligament parameters (26 parameters total, Table 3) in Abaqus for the simulation of a given calibration target. Using the Abaqus API, custom Python scripts extracted simulation results, including kinematics. The squared error between simulated GS kinematics and experimental GS kinematics was calculated for each kinematic DOF. This was repeated for each of the given calibration targets to calculate the optimization cost function (described below). The optimization process occurred in two phases for each knee model. First, a particle swarm global optimization (Kennedy and Eberhart, 1995) narrowed the search space to the location of the most likely global minimum. Then, using the ligament parameters at this approximate minimum as an initial point, a Nelder-Meade Simplex solver determined the true local minimum around this point (Nelder and Mead, 1965). The approximate number of iterations to reach a minimum was 750 for the particle swarm optimization and 500 for the Nelder-Meade Simplex solver. While the exact time required to complete an iteration for each model calibration depended on the number of calibration targets and elements within the model, the average clock time was approximately 210 s per iteration. Therefore, the overall time to complete calibration for each knee model was approximately 73 h (single Intel Xeon Gold 6134 CPU @ 3.2 GHz).

**FIGURE 6:**
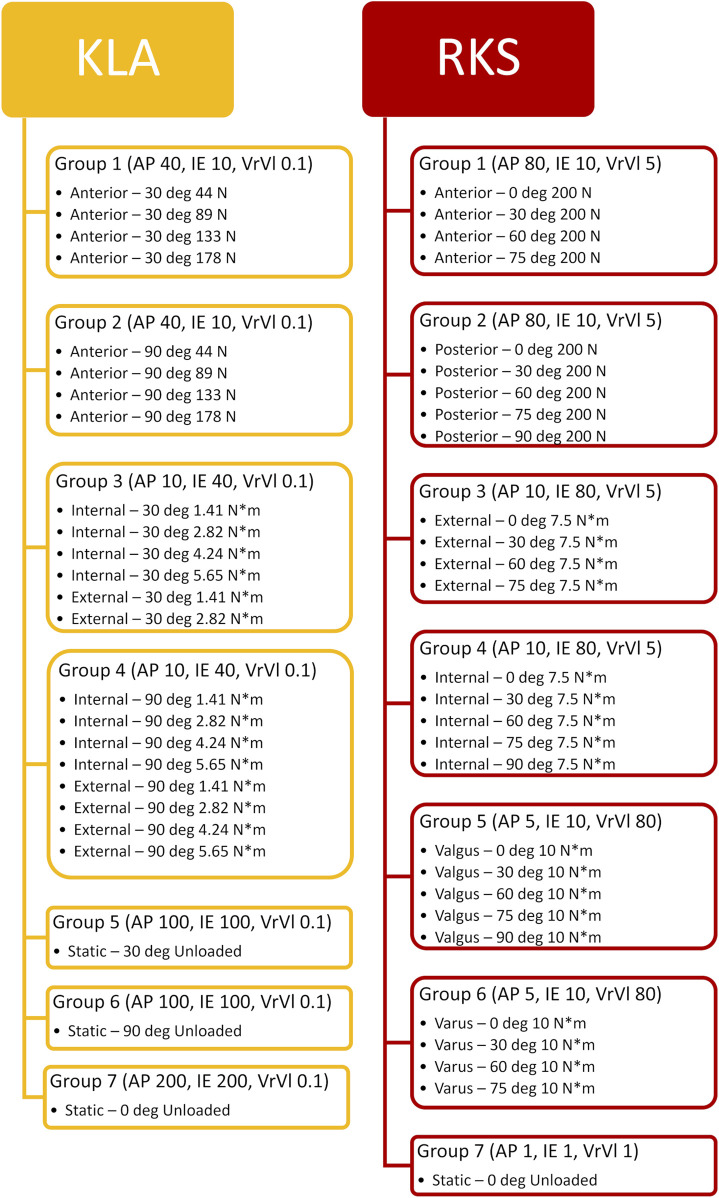
Workflow of the corresponding laxity groups created by grouping together similar trials and experimental laxity points. Values in parentheses represent the corresponding weight applied to errors between the simulation predicted kinematics and the actual experimentally observed kinematics for that DOF.

The optimization process minimized a cost function consisting of the squared error between the measured and simulated calibration targets and a penalty term. Trials were grouped in similar categories (e.g., anterior laxity at 30° of knee flexion for multiple loads), and the 75th percentile of the root mean squared error (RMSE) for each DOF of a given group was calculated and normalized to the range between the minimum and maximum observed for each kinematic DOF across all experimental results. Targets were grouped together (Figure 6) to bias the optimization across the range of flexion angles and DOF (rather than, for instance, AP at 30° at 10 N, 20 N, 30 N, and 40 N). The normalized errors for each DOF were then scaled by chosen scalar weights and summed across all categories into a total cost. This weighting allowed selected DOF to be more emphasized based on the primary DOF for a given laxity trial (AP for anterior at 30°, IE, for internal at 90°, etc.) while allowing for secondary DOF (IE, for anterior at 30°, AP for internal at 90°, etc.) to also be included with less emphasis. Additionally, a penalty term squared the cost if any trials reached joint limits on the SI and ML DOF. The penalty improved optimization speed by quickly guiding the search away from unrealistic solutions. An example calculation from a single iteration of the optimization for the Specimen 2 model calibrated to KLA targets is included in the Supplementary Material as a spreadsheet file.

### 2.6 Model performance comparison and validation

#### 2.6.1 Ligament parameters

The resulting ligament material property parameters (reference strain and stiffness) for each calibrated model were compared to evaluate the differences between the KLA and RKS models, and between specimens. Values reported are the true parameters calibrated for each model. However, because of the large range of resulting material parameters, particularly between reference strains and stiffnesses, percentage differences were also calculated in each case to simplify model and specimen comparisons.

#### 2.6.2 AP laxity

To compare AP laxity of the models, AP loads of 133 N and −133 N were applied to each model at 30, 60, and 90° of knee flexion. A magnitude of 133 N (30 lbf) load was chosen as it is commonly used when evaluating knee laxity clinically (Un et al., 2001; Starkel et al., 2014). Simulation of AP laxity was performed as described above with the addition of a third simulation step in Abaqus/Explicit that linearly increased and decreased the AP loading between 133 N and −133 N. The GS kinematics for the AP direction were recorded and root-mean-squared difference (RMSD) was calculated between the models calibrated with laxity measurements from the KLA and RKS.

#### 2.6.3 Passive flexion

Passive flexion was simulated by applying zero loads for all DOF and prescribing knee flexion angle from 0 to 90°. A two-step Abaqus/Explicit simulation was performed as described above. Results were obtained for the predicted passive flexion kinematics of AP, IE, and VrVl vs. knee flexion angle. Results were compared to the experimental values measured for passive flexion, namely, Intact Leg Experimental and Dissected Knee Experimental data.

#### 2.6.4 Pivot shift

To compare the models during complex motions, a simulated pivot shift test was performed. The pivot shift test is a clinical evaluation that aims to determine the stability of the knee as a means of predicting possible anterior cruciate ligament (ACL) injury (Matsushita et al., 2013). Following previous work, a pivot shift was simulated by placing the knee in 30° of flexion and applying an 8 N*m valgus torque combined with a simultaneous 4 N*m internal torque (Schafer et al., 2016; Thein et al., 2016). A two-step simulation was performed as described above. Model kinematics were recorded for the KLA and RKS models for both specimens. In addition, an ACL-deficient pivot shift was simulated for all models and calibrations. The ACL-deficient condition was simulated by creating a parameter that controlled the presence of a failure in the connectors representing the ACL for both the anteromedial and posterolateral bundles (connector failure in Abaqus). A parameter equation was created that caused an immediate failure of the ACL connectors in the first simulated time increment of the model. Following the failure of the connectors, the simulation progressed through the remaining time steps as if the ACL was not present, simulating a complete tear of the ligament. Kinematics and ligament forces were compared between models for the intact and ACL-deficient conditions.

## 3 Results

### 3.1 Ligament parameters

The calibrated material properties for ligament reference strain and stiffness are reported in Table 4. The smallest reference strain on average was observed in the dMCL with a value of 0.85 and the largest observed in the PCAP_M with a value of 1.17. The smallest stiffness on average was observed in the PFL with a value of 51.9 N/mm and the largest observed in the dMCL with a value of 138.9 N/mm. Percent differences between stiffnesses were approximately 4 times greater than the percent differences observed for reference strains on average. The percent differences for the reference strains were lower between models (KLA vs. RKS) as compared with between specimens, with the average differences found of 4.6% and 12.3% for inter-model (KLA vs. RKS) and inter-specimen (Specimen 1 vs. Specimen 2), respectively. The same effect was observed for stiffness where the average difference was 14.3% and 55.8% for inter-model and inter-specimen, respectively.

**TABLE 4:** Optimized ligament parameters for KLA and RKS models for both specimens, respectively. KLA is model calibration using data from the knee laxity apparatus. RKS is model calibration using data from the robotic knee simulator. X1 represents the first design variable, X2 represents the second design variable, and so on.

Material parameter	Design variable	Ligament	Specimen 1		Specimen 2	
			KLA	RKS	KLA	RKS
Reference Strain	X1	ACL_AM	1.14	1.07	1.16	1.14
	X2	ACL_PL	1.00	1.00	1.21	1.23
	X3	LCL	0.94	0.96	0.76	1.06
	X4	sMCL_A	0.79	0.80	1.05	0.94
	X5	sMCL_M	0.90	0.91	1.00	0.92
	X6	sMCL_P	0.84	0.85	0.98	0.90
	X7	dMCL	0.95	0.94	0.79	0.74
	X8	PCL_AL	0.91	0.92	0.88	0.89
	X9	PCL_PM	0.93	0.94	0.87	0.94
	X10	ALS	1.00	1.00	0.76	0.82
	X11	PFL	0.99	1.14	1.15	1.15
	X12	POL	1.06	1.00	0.92	0.90
	X13	PCAP_M	1.25	1.25	1.08	1.11
	X14	PCAP_L	1.17	1.22	1.12	1.14
Stiffness (N/mm)	X15	ACL_AM	79.41	68.53	103.85	79.21
	X16	ACL_PL	114.56	115.94	50.19	58.17
	X17	LCL	90.35	92.57	111.76	125.14
	X18	sMCL	95.58	102.06	85.05	40.65
	X19	dMCL	157.53	153.26	111.79	133.03
	X20	PCL_AL	72.87	77.64	64.25	63.70
	X21	PCL_PM	82.13	85.37	30.17	30.14
	X22	ALS	120.16	114.86	53.58	49.17
	X23	PFL	19.90	32.56	89.52	65.68
	X24	POL	73.99	65.06	71.60	66.43
	X25	PCAP_M	71.12	74.38	72.41	71.49
	X26	PCAP_L	61.58	60.51	70.00	67.23

### 3.2 AP laxity

**FIGURE 7:**
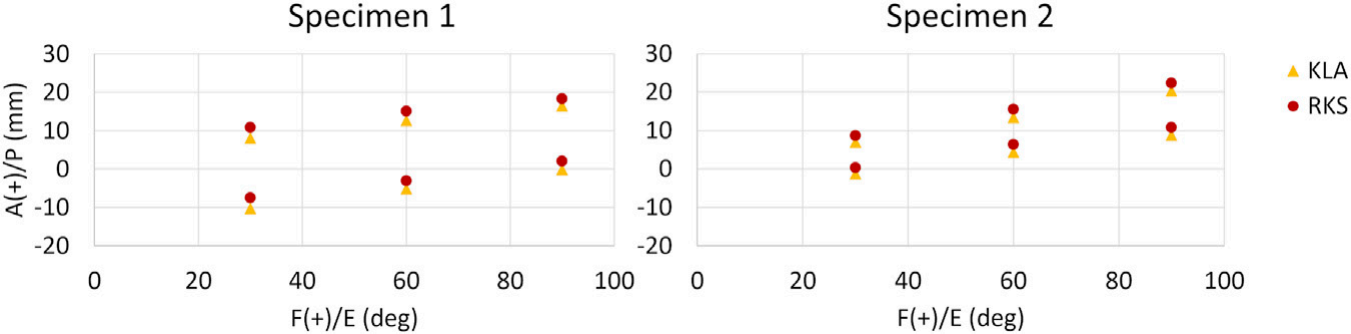
AP GS kinematics from 133 N anterior (top points) and posterior (bottom points) tibial load at three knee flexion angles for all models and both specimens. (KLA) Simulated AP laxity from model calibrated with data from the knee laxity apparatus. (RKS) Simulated AP laxity from model calibrated with data from the robotic knee simulator.

Predicted AP translation in response to 133 N anterior and posterior load was similar between the models calibrated with KLA and RKS measurements (Figure 7). The RMSD in the anterior direction between the KLA and RKS models was 2.38 mm and 1.94 mm for Specimen 1 and Specimen 2, respectively. RMSD in the posterior direction was 2.45 mm and 1.90 mm. The ACL was the most loaded ligament for the anterior laxity trials at all flexion angles for all models (Table 5). For the posterior direction, the PFL and POL were loaded for both models in Specimen 1. The PCL was the most loaded ligament for Specimen 2. The RMSD of ligament loads between the KLA and RKS models was 10.3 N and 11.4 N for Specimen 1 and 2, respectively.

**TABLE 5:** Predicted ligament loads from 133 N anterior and posterior tibial load at three knee flexion angles for KLA vs. RKS models for both specimens. KLA is model calibration using data from the knee laxity apparatus. RKS is model calibration using data from the robotic knee simulator. All loads recorded under 5 N are represented as a “-”.

Laxity direction	Knee angle (deg)	Specimen	Model	ACL (N)	ALS (N)	LCL (N)	MCL_S (N)	MCL_D (N)	POL (N)	PCAP (N)	PCL (N)	PFL (N)
Anterior	30	1	KLA	163.4	-	-	-	14.1	-	-	-	-
Anterior	30	1	RKS	173.1	-	-	-	16.5	-	-	-	-
Anterior	30	2	KLA	248.5	-	-	-	-	-	-	-	83.3
Anterior	30	2	RKS	246.9	-	60.1	-	-	-	-	-	66.6
Anterior	60	1	KLA	161.4	13.2	-	-	21.5	-	-	-	-
Anterior	60	1	RKS	146.3	38.3	-	-	40.2	-	-	-	-
Anterior	60	2	KLA	203.8	-	-	18.1	-	-	-	-	34.0
Anterior	60	2	RKS	206.3	-	12.4	-	-	-	-	-	35.9
Anterior	90	1	KLA	120.3	58.2	-	-	57.6	-	-	-	-
Anterior	90	1	RKS	85.2	93.5	-	-	84.7	-	-	-	-
Anterior	90	2	KLA	194.0	16.9	-	32.3	-	-	-	-	13.0
Anterior	90	2	RKS	195.0	8.9	-	23	-	-	-	-	-
Posterior	30	1	KLA	-	-	-	-	-	73.4	-	-	89.9
Posterior	30	1	RKS	-	-	-	-	-	65.3	-	-	75.2
Posterior	30	2	KLA	-	-	-	14.1	-	-	-	121.8	80.6
Posterior	30	2	RKS	-	-	33.7	-	-	-	-	128.9	73.1
Posterior	60	1	KLA	-	-	-	-	-	60.0	-	-	65.6
Posterior	60	1	RKS	-	-	-	-	-	56.1	-	-	64.0
Posterior	60	2	KLA	-	-	-	-	-	-	-	138.2	51.6
Posterior	60	2	RKS	-	-	-	-	-	-	-	130.0	50.3
Posterior	90	1	KLA	-	-	-	-	-	66.4	15.2	-	58.7
Posterior	90	1	RKS	-	-	-	-	-	41.5	33.4	-	60.2
Posterior	90	2	KLA	-	-	-	-	-	-	-	166.8	-
Posterior	90	2	RKS	-	-	-	-	-	-	-	155.5	32.8

### 3.3 Passive flexion

**FIGURE 8:**
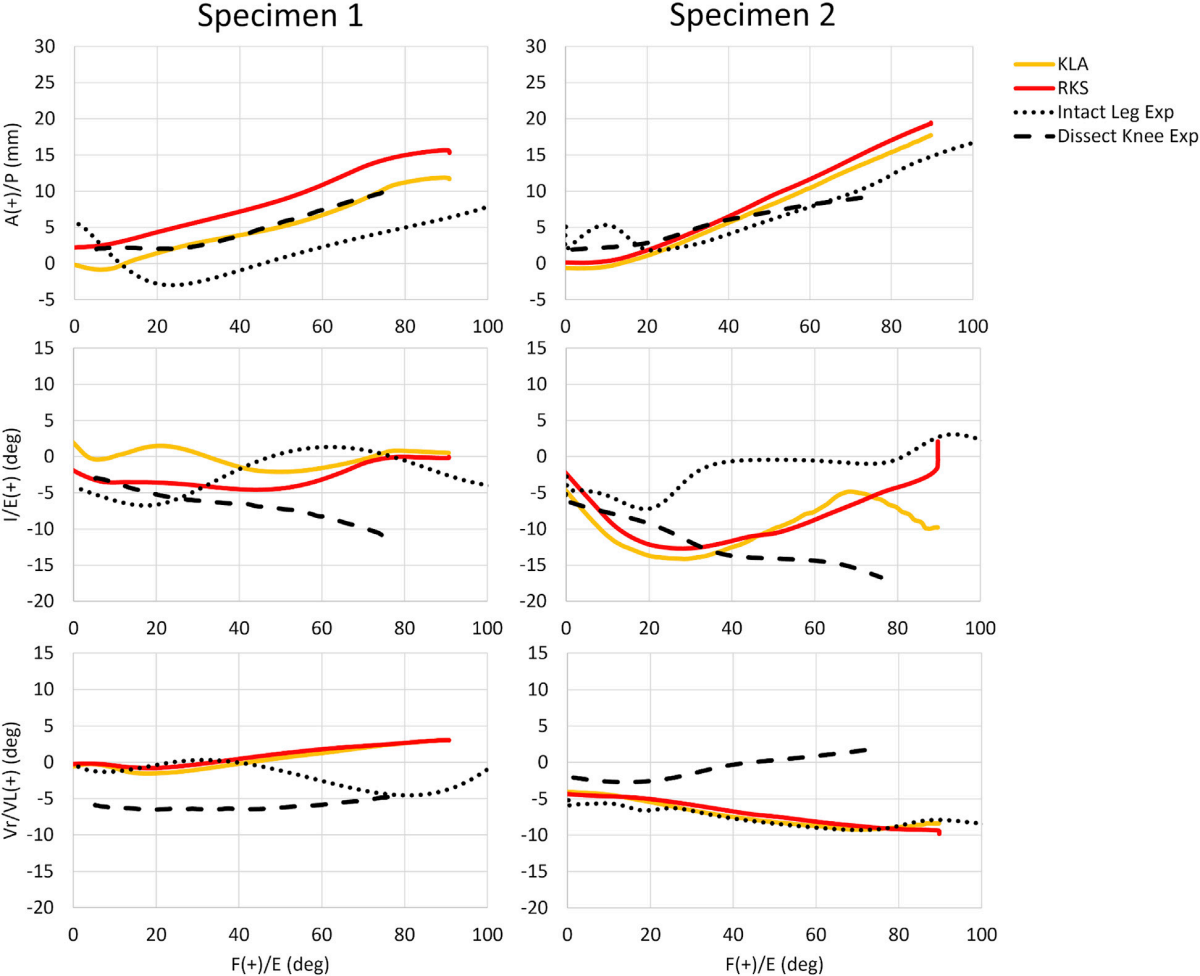
Passive knee flexion GS kinematics for both specimens and all model simulations vs. experimental measures for anterior-posterior (AP), internal-external (IE), and varus-valgus (VrVl) vs. knee flexion angle. (KLA) Simulated passive knee flexion from calibration of model with data from the knee laxity apparatus. (RKS) Simulated passive knee flexion from calibration of model with data from the robotic knee simulator. (Intact Leg Exp) Experimental passive knee flexion collected via manual manipulation of the limb by the experimenter through the range of motion for the fully intact lower-limb. (Dissect Knee Exp) Experimental passive knee flexion collected via a robotic knee simulator no-load motion on the dissected knee.

Predicted AP translation of the tibia was similar in magnitude and trend for both models and specimens during simulated passive flexion (Figure 8). While the prediction of IE, and VV was similar for both models and specimens, some differences were noted. The RMSD between the KLA and the RKS model was 3.5 mm, 2.6°, and 0.4° for AP, IE, and VrVl, respectively, for Specimen 1 and 1.1 mm, 1.2°, and 0.3° for Specimen 2. The kinematics for the experimental datasets varied even for the same specimen. The differences between experimental curves were more significant for the rotational DOF (IE, and VrVl) as compared to the translation DOF (AP). Moreover, the differences between experimental curves were larger than the differences between the model predictions, with the model predictions generally falling within the envelope of motion between experimental curves. Kinematics for the IE, DOF in specimen 2 for the models diverged for flexion angles greater than 70°.

### 3.4 Pivot shift

Kinematics during the simulated pivot shift were within 2.6° and 2.8 mm between the KLA and RKS models for all simulations for rotations and translations, respectively (Table 6). The simulated ACL-deficient condition for Specimen 2 was an exception, where both the RKS and KLA models predicted a dislocation. All models predicted increases in tibial anterior translation and valgus rotation for the ACL-deficient condition relative to the intact model. All models predicted an increase in the anterolateral structure (ALS) ligament load for the ACL-deficient condition compared with the intact condition (Table 7). Ligament loads for the collateral ligaments (LCL and MCL) were zero for both models of Specimen 1 but were non-zero for Specimen 2. In both the KLA and RKS models for Specimen 2, the sum of the LCL and MCL ligament loads decreased for the ACL-deficient condition relative to the intact condition, with the KLA model decreasing by 67.4 N, while the RKS model decreased by 53.2 N.

**TABLE 6:** Predicted GS kinematics during simulated pivot shift at 30° of knee flexion with 8 N*m valgus torque and 4 N*m internal torque. KLA is model calibration using data from the knee laxity apparatus. RKS is model calibration using data from the robotic knee simulator. Models with an “*” denote a simulation with a predicted dislocation between the femur and tibia wherein the kinematics reported may be unreliable.

Specimen	ACL condition	Model	F (+)/E (deg)	Vr/Vl (+) (deg)	I/E (+) (deg)	M/L (+) (mm)	A (+)/P (mm)	S (+)/I (mm)
1	Intact	KLA	30.9	2.7	−13.3	0.4	3.9	−17.3
1	Intact	RKS	30.9	5.3	−15.0	−0.2	4.7	−18.3
1	No ACL	KLA	30.9	5.9	−11.3	−0.7	8.1	−18.4
1	No ACL	RKS	30.9	6.8	−13.6	−0.1	7.0	−18.9
2	Intact	KLA	29.9	−3.1	−28.8	0.0	4.6	−28.8
2	Intact	RKS	29.9	1.6	−31.6	−2.2	1.8	−30.5
2	No ACL	KLA*	29.9	3.7	−25.1	−2.1	21.6	−24.4
2	No ACL	RKS*	29.9	12.2	−19.8	−11.4	23.1	−24.8

**TABLE 7:** Predicted ligament loads during simulated pivot shift at 30° of knee flexion with 8 N*m valgus torque and 4 N*m internal torque. KLA is model calibration using data from the knee laxity apparatus. RKS is model calibration using data from the robotic knee simulator. Models with an “*” denote a simulation with a predicted dislocation between the femur and tibia wherein the loads reported may be unreliable.

Specimen	ACL condition	Model	ACL (N)	ALS (N)	LCL (N)	MCL_S (N)	MCL_D (N)	POL (N)	PCAP (N)	PCL (N)	PFL (N)
1	Intact	KLA	46.9	121.8	0.0	0.0	0.0	103.1	0.0	0.0	0.0
1	Intact	RKS	17.5	145.9	0.0	0.0	0.0	86.2	0.0	0.0	0.0
1	No ACL	KLA	0.0	163.7	0.0	0.0	0.9	85.6	0.0	0.0	0.0
1	No ACL	RKS	0.0	159.3	0.0	0.0	5.2	81.3	0.0	0.0	0.0
2	Intact	KLA	175.1	0.3	0.0	215.4	0.0	0.0	0.0	0.0	93.1
2	Intact	RKS	141.6	28.7	190.2	88.6	0.0	0.0	6.9	95.7	0.4
2	No ACL	KLA*	0.0	187.3	0.0	148.0	0.0	0.0	29.1	0.0	46.9
2	No ACL	RKS*	0.0	271.3	41.1	184.5	0.1	0.0	0.6	−0.1	0.1

## 4 Discussion

Recent calls for more personalized approaches to medicine, including digital twins and *in silico* clinical trials, have prompted an increased demand for computational models of living people. Personalized knee models could be used in conjunction with existing surgical planning tools to better predict and understand the short- and long-term outcomes of various treatment options. However, the necessary tools to obtain measurements of the living knee for model calibration are limited. While knee laxity is a routine clinical evaluation, these measurements have historically been insufficient to calibrate models with useful accuracy. Furthermore, while recent work has examined the effects of modeling methodologies on model performance, the impact of the data used to build and calibrate models has received little attention. This study investigated the effects of experimental inputs on model predictions following model calibration using knee laxity measurements from *in vivo* (KLA) and *in vitro* (RKS) methods. Specimen-specific FEM of the knee were developed and calibrated, and model performance was compared. Our results showed that accurate model calibration may be achieved using measurements available from living subjects.

Whether calibrated from KLA or RKS measurements, the models captured the distinctly different behavior of the two knee specimens. The differences in calibrated material parameters were greater inter-specimen as compared to inter-model (Table 4). This highlights that the models built using different calibration data (KLA vs. RKS) can capture the unique material behavior of each specimen. However, for almost every ligament, we observed that the percent differences for reference strain were smaller than those for stiffness. Previous work from Baldwin et al. used Monte Carlo and Advanced Mean Value (Wu et al., 1989) analyses to determine the relative importance of model parameters on knee joint laxity (Baldwin et al., 2009). They found that the reference strain was frequently more important than stiffness for accurate recreations of joint laxity. This may explain why reference strain values in our models were more similar than the overall ligament stiffness, because accurate recreation of joint motion, particularly in loaded conditions, was more sensitive to reference strain rather than stiffness.

For each specimen, results were similar for both the passive flexion and the AP laxity simulations despite the differing calibration targets from each data source (Figures 5, 6). The RKS measurements provided laxity data for 3 DOF at four knee flexion angles while the KLA *in vivo* measurement device provided laxity in only 2 DOF at two knee flexion angles. Still, the predictions of the AP displacement in response to applied load were similar and unique to each specimen, with similar ligament loading observed for all AP conditions (Table 5). The resulting errors between model predicted kinematics for the AP conditions across both specimens were less than 2.5 mm and 2.0 mm for anterior and posterior laxity, respectively (Figure 7); these errors are within the minimum detectable change (MDC) for AP laxity reported from other *in vivo* knee laxity devices, with MDCs ranging from 1.1 mm to 4.5 mm (Mouton et al., 2015; Smith et al., 2022; Imhauser et al., 2024). Notably, posterior knee laxity was accurate despite no posterior loading targets in the KLA model calibration. Additionally, model predictions of AP laxity were similar at 60° of knee flexion, despite the KLA model not having this flexion angle in the calibration targets for either specimen. Furthermore, the low RMSD between KLA and RKS models during passive flexion (flexion free of dynamic loads) suggests that both models predict nearly the same kinematics despite not being calibrated to passive data. For both specimens, differences between calibration with RKS or KLA data were within the errors reported for passive flexion from another study, wherein knee models were built from the same experimental data but with different modeling workflows (Andreassen et al., 2023). These results help demonstrate that laxity measurements from *in vivo* techniques, such as those from the KLA, can provide sufficient targets for model calibration in subject-specific modeling.

The models calibrated from RKS and KLA predicted similar kinematics for a simulated pivot shift test. The maximum differences between model predictions were within 2.6° and 2.8 mm for rotation and translation, respectively. Inter-specimen differences in kinematics were far greater than inter-model differences in both ACL-intact and deficient conditions (Table 6). Additionally, both the KLA and the RKS models predicted the same dislocation behavior in Specimen 2 for the ACL-deficient condition. In agreement with Thein et al., force in the ALS of the knee increased without the ACL in all models (Thein et al., 2016). This demonstrates that models calibrated using data acquired with *in vivo* methods can make meaningful predictions beyond the calibration data, including dislocation behavior. Still, while similar ligament loads were observed between the KLA and RKS models for the ACL-intact and deficient conditions, differences remain. For Specimen 2, in the intact condition with KLA calibration, the LCL force was 0.0 N and the superficial MCL force was 215.4 N; in contrast, the RKS calibration predicted the LCL force to be 190.2 N and the superficial MCL force to be 88.6 N (Table 7). In addition, there are portions where the reference strain of a ligament for Specimen 1 is greater for the KLA model compared to value for Specimen 2, but the opposite scenario is found for the RKS model between the two specimens, such as for the LCL (Table 4). Together, these results highlight that while models may yield similar joint-level force-displacement behavior from different ligament material properties, the resulting ligament loads may be variable. Similarly, recent work by Theodorakos et al. showed that model calibration is sensitive to initial conditions for the material properties used for model calibration (Theodorakos and Andersen, 2024). They showed that different initial conditions resulted in different material properties following model calibration and different ligament forces despite small overall kinematic and kinetic differences at the joint-level. Thus, subject-specific models created to predict *in vivo* ligament loads may not provide accurate results using joint-level calibration alone. Additional calibration constraints or penalties informed by subject-specific information may be necessary to drive the calibration to a set of material properties that ensure feasible predictions of ligament loads. These penalties could integrate physiologically relevant phenomena that further enhance the realism of the results. For example, the inclusion of an overall muscle activation penalty based on various expenditure and muscle synergy laws is frequently used in musculoskeletal modeling to distribute muscle activation (Delp et al., 2007). These types of additional penalties have been shown to distribute muscle activation more closely to experimentally observed distributions from electromyography than from force-displacement optimization alone (Michaud et al., 2020). Similar modeled penalties, such as minimizing overall ligament strain or force, could be implemented in these model calibration workflows, which may better explain the underlying physiology and improve model predictions of ligament forces.

While the FEA results from the KLA and RKS models are highly consistent with each other overall, there are some inconsistencies with experimental measurements in passive flexion. In Figure 8, the KLA and RKS model results are similar to the experimental measurements in AP, IE, and VrVl at full extension, and in AP kinematics as the knee flexes and largely fall within the envelope of passive motion of the experimentally observed data. However, with increasing knee flexion, IE experimental trends diverge. Likewise, the model results and experimental measurements diverge in VrVl with increasing flexion. These differences primarily in IE and VrVl occur for two reasons. First, the differences in kinematics observed experimentally highlight the uncertainty in passive knee motion under small input loads. Blankevoort et al. described how small changes in loads result in large differences in joint kinematics (Blankevoort et al., 1988), particularly when the overall joint compression is small. Furthermore, internal and external rotation range of motion at the knee naturally increases as the knee flexes, especially beyond 30° of flexion (Zarins et al., 1983). Second, there were unmeasured loading differences in the Intact Leg Experimental data and the Dissected Knee Experimental data. In the Intact Leg Experimental data, fully intact lower limbs were manually moved by cable through knee flexion to extension, while in the Dissected Knee Experimental, passive knee flexion of the dissected knees was robotically controlled. Meanwhile, simulation of passive flexion with the KLA and RKS models was fully unloaded, with only knee flexion kinematically driven and lacking significant modeled soft-tissue structures. The large envelope of passive motion between the experimental curves highlights the need for near-identical conditions between situations, particularly with small loads, even for the same knees. For these reasons, the kinematics of the KLA and RKS models are similar but do not always match the trends of experimental data. These results highlight that the inherent variability and sensitivity of passive flexion to loading conditions, and the presence of soft-tissue structures, make it potentially unsuitable for validation.

The larger point of Figure 8 is to demonstrate that the models behave very similarly to one another, despite using different data sources, and are close to the overall envelope of motion observed experimentally. Any discrepancies remaining are likely limitations of the “art” of modeling itself, due to the lack of certain structures and the inability to recreate the exact loading conditions of the original experiment. Even so, passive flexion was investigated here primarily to enable comparison to prior work that evaluated the impact of modeling calibration workflow on model performance (Andreassen et al., 2023); in particular, with this work using the same modeling workflow and steps as the Team DU model from that work. In the aforementioned KneeHub project, five different groups performed model development and calibration on specimen-specific models using each group’s chosen modeling and calibration workflow, but with the same experimental data (Erdemir et al., 2019; Rooks et al., 2021; Andreassen et al., 2023). Using raw data reported from that work, average inter-model RMSD in passive flexion were as high as 6.2 mm, 14.9°, and 6.8° for AP, IE, and VrVl, respectively; considerably higher than the maximum values found herein of 3.5 mm, 2.6°, and 0.4°, for AP, IE, and VrVl, respectively. Moreover, Andreassen et al. found an average 10% difference in inter-model reference strain between the five modeling strategies, compared with the 4.6% inter-model observed here. These differences suggest that when comparing passive flexion, the modeling workflow, or “art”, has as great an influence on model performance as measurement methodology and the specific targets used for calibration. Moreover, it is important to consider that for *in vivo* modeling, it is impractical to record a truly passive joint motion even with anesthesia, making it a poor kinematic target for ligament material property calibration and benchmarking, apart from full extension. Thus, while the consistent results in the three conditions we tested (passive, AP laxity, and pivot shift) support the use of calibration with *in vivo* experimental measurements, the calibration of models to the living knee should include measurements that reflect the intended context of use.

This study has limitations. The first limitation is the small number of knee specimens utilized, which limited the power of the study to investigate subject variation and make generalizable claims. The results found should be considered as indicative of potential but insufficient to establish scientific consensus. Still, the sample size of two specimens was comparable to other studies of subject-specific knee modeling (Kia et al., 2016; Ali et al., 2017; Razu et al., 2023) and this work highlights the promise of using recently available tools *in vivo* as a means to calibrate knee models. However, future work should include larger sample sizes and specimen variation to ensure these initial results hold in the presence of greater variability. A second limitation is the applicability of the laxity measurements made herein to those performed in living individuals. In measuring knee laxity *in vivo*, there is the potential for physiological factors such as passive muscle tone, coactivation, spinal reflexes, pathology, and other contributions to muscle force that influence the amount of knee laxity measured from the passive structures alone. Previous work has shown that laxity in the knee during an anterior drawer test increases in patients under anesthesia compared to when awake (Matsushita et al., 2013). Future work using living individuals should include methods to reduce the possibility of muscle-reduced knee laxity by employing muscle stretch-relaxation techniques (Osternig et al., 1987) or fatiguing muscle contractions (Nawata et al., 1999), which has been shown to increase knee laxity. In addition, future studies involving living subjects should include methods to determine the relative activation of muscles such as electromyography (EMG). Another related limitation is that models were developed using CT and white-light surface scans, which would be impractical *in vivo*. We chose to use the surface scan combined with CT, as compared to MRI or statistical methods, to ensure the highest possible accuracy for identifying ligament attachment sites and minimize the variability this would cause on the results and analysis. Moreover, other groups have focused on developing accurate methods of predicting attachment sites, with promising results (Pillet et al., 2016; Malbouby et al., 2025). Future work should investigate the impacts of all *in vivo* measurements to show how these effects may compound. Another limitation is that while the RMSD between model predictions herein is small, within 2.5 mm for AP laxity, and for passive flexion less than the errors found in the KneeHub project (Andreassen et al., 2023), regions of higher flexion in Figure 8 show differences between model performance and measurements under small loads. While we tested three conditions to demonstrate the efficacy of ligament material property calibration using methods and measurements available to living subjects, the differences emphasize the importance of calibration to measurements relevant to the context of use when modeling the living knee. For some applications, the current accuracy may not be sufficient. The final limitations are the representation of cartilage as linear elastic isotropic and the lack of a meniscus or patella in the model. The cartilage model was simplified to decrease computational burden, and previous work has shown that models utilizing linear-elastic isotropic representations of cartilage can accurately predict experimentally measured joint contact (Kiapour et al., 2014). Furthermore, as this work aimed to examine the kinematics of the knee and not contact patterns or stress, the choice of cartilage material property likely had little effect on the observed results. Still, future work should investigate if there are significant effects of the choice of cartilage material models on ligament material calibration. The models did not include the menisci, in line with other studies (Farshidfar et al., 2022), as inclusion of the menisci has been shown to have little effect on the kinematics of the knee at less than 90° of knee flexion (Amiri et al., 2006). Still, for certain contexts of use, inclusion of menisci is crucial. Additionally, the models did not include a patella, in line with other studies (Harris et al., 2016; Farshidfar et al., 2022; Rooks et al., 2022). The primary objective of this work was to recreate tibiofemoral kinematics, and therefore, the patella was not included. Still, the lack of a patella and meniscus may have impacted some of the results herein, such as in the passive flexion curves in Figure 8. Future studies should investigate if the presence of menisci and patella influences ligament material calibration.

In summary, this study reported small errors for the models calibrated to data measured with a laxity measurement apparatus, designed for the living knee, compared with models calibrated to data measured with a robotic knee joint simulator. The viability of using knee laxity measurements in future calibration of living subjects was demonstrated by close agreement with knee calibration using measurements from cadaveric testing. Still some differences were observed between models, particularly in predicted loads, suggesting that modeling workflow has as great an influence on model performance as measurement methodology and the specific targets used for calibration. Overall, the workflows and optimization strategies described here can act as a basis for future subject-specific modeling and the development of digital twins. The experimental data, models, results, and tools created are publicly available to encourage model reproducibility.

## Data Availability

The datasets presented in this study can be found in online repositories. The names of the repository/repositories and accession number(s) can be found below:https://simtk.org/projects/in_vivo_valid, https://doi.org/10.5061/dryad.zkh1893gw, https://doi.org/10.5061/dryad.zcrjdfnpv, https://doi.org/10.5281/zenodo.10416663, https://doi.org/10.5281/zenodo.14664715, https://zenodo.org/records/10521352.

## Supplementary material

SUPPLEMENTARY TABLE S1 An example calculation of the overall cost calculation taken froma single iteration of the model optimization. File shows how the cost is calculated across each individuals simulation and then grouped together based on the corresponding laxity groups.

SUPPLEMENTARY DATA SHEET S1 Supplementary information containing additional descriptions of model development, cartilage calibration steps, and model verification.
