# Supplementary material for: Validating subject-specific knee models from in vivo measurements
Source: Front Bioeng Biotechnol. 2025 Aug 14;13:1554836. doi: 10.3389/fbioe.2025.1554836 (PMC12391196; doi:10.3389/fbioe.2025.1554836)
Supplement: Supplementary file 1 [file DataSheet1.pdf]

Supplementary Material

Validating Subject-Specific Knee Models from In Vivo Measurements

Thor E. Andreassen[1,2]*, Donald R. Hume[1], Landon D. Hamilton[1], Stormy L. Hegg[1]
Sean E. Higinbotham[1], Kevin B. Shelburne[1]

[1]Center for Orthopaedic Biomechanics
Department of Mechanical and Materials Engineering
University of Denver
Denver, CO, USA

[2]Assistive and Restorative Technology Laboratory
Department of Physical Medicine and Rehabilitation,
Mayo Clinic
Rochester, MN, USA

*Correspondence:
Corresponding Author
thor.andreassen@du.edu

1 Full Analysis Overview

The full model workflow used to capture data, build models, calibrate material properties (cartilage and ligament) and the final model result comparison, is shown below in Figure 1. Models were built from segmentation of computed tomography (CT) scans of the lower extremities of the specimens. These geometries combined with surface scans of the bones taking during dissections were the basis for the finite element analysis (FEA) models built. Ligament structures were defined based on anatomical descriptions of the geometries. Individual fibers were chosen based on fitting an ellipse to the ligament sites and spanning the major axis. Target laxity points were chosen from the obtained specimen-specific laxity measurements captured with the knee laxity apparatus (KLA) and the robotic knee simulator (RKS). Model verification was performed to ensure adequate size elements for cartilage geometry, and then a rigid-rigid cartilage model was created and calibrated to match the results of the deformable cartilage model. Laxity measurements were used to calibrate individual ligament material properties as described in the main article. Kinematics and ligament loads were observed for the models under different anterior-posterior laxity conditions, passive flexion, and a simulated pivot shift test.

The details of the mesh verification, ligament fiber ellipse procedure, and the cartilage material calibration are provided below.

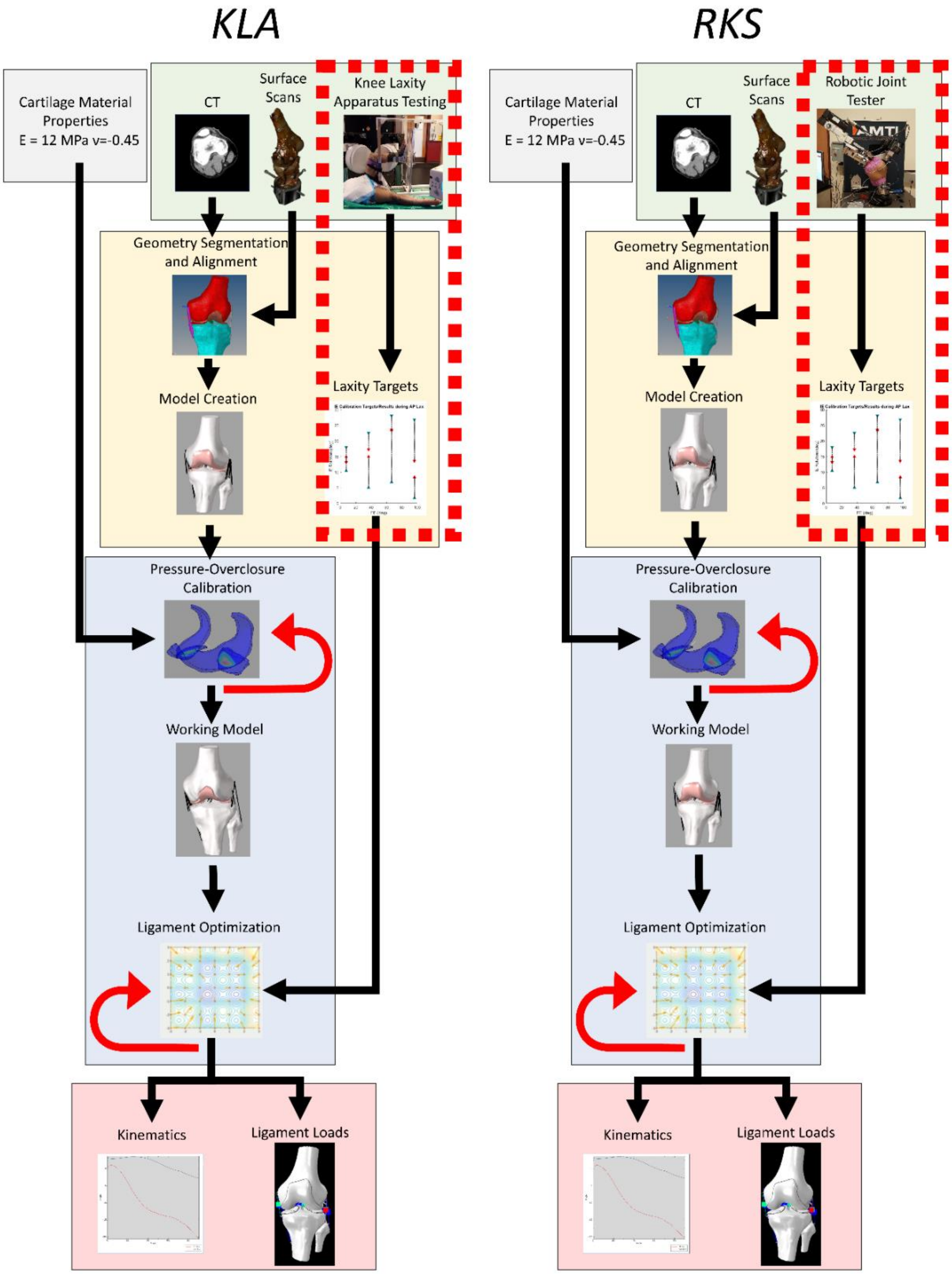

Figure 1: Workflow of model development for KLA and RKS Models. (Gray) is the cartilage material property from literature. (Green) Input data from experimental collection, namely CT scans, surface scans, and knee laxity data. (Yellow) Model creation and preprocessing of laxity target values. (Blue) Cartilage and ligament material property calibration. (Red) Output model validation for kinematics and ligament loads. (Red Dashed Region) Portion that is individual to each model (KLA vs. RKS) based on difference in model data. (Black Arrows) Describe single steps. (Red Arrows) Optimization steps.

2 Mesh Verification

To verify the size of the elements used for cartilage, a mesh size convergence study was performed according to recommendations from Anderson et al. (Anderson et al., 2007). A set of femoral and tibial cartilage geometries with hexahedral elements with nominal sizes ranging between 1.0 and 0.5 mm were created in HyperMesh (Altair Engineering Inc., Troy, MI). Finite element analysis (FEA) models were created in Abaqus Explicit (Dassault Systems, France) using the cartilage geometries of corresponding element sizes. Rigid surfaces were created for the tibial and femoral cartilage boney surfaces. The tibial cartilage boney surface was completely fixed in all degrees of freedom (DOF, boundary encastre in Abaqus). The femoral cartilage boney surface was placed in displacement control for all DOF except for the superior-inferior (SI) direction which applied a linearly ramping compression in load control. Model contact was defined for the articular surfaces of the tibial and femoral cartilage, and models simulated contact of the femoral cartilage into the tibial contact in pure compression.

Contact area, maximum contact pressure, net SI femoral displacement, and maximum von Mises stress were recorded at 250 and 500 N of compression. The convergence study showed that cartilage displacement, von Mises stress, and contact area changed by less than 1% between elements with 1.0 mm and 0.5 mm size (Table 1). This verifies that elements with at least 1.0 mm element sizes, would be adequate for the analysis in the main study, where contact metrics were not the main output of interest.

For the main study, to achieve a minimum of 4 elements through the thickness of the cartilage while maximizing element quality metrics, different cartilage geometries were meshed with slightly different approximate element sizes; however, all cartilage geometries had target element lengths at the largest between 1.0 and 0.5 mm.

Table 1: Mesh convergence study of femoral and tibial cartilage element size. Relative error is the percent difference between the 1 mm element and the 0.5 mm element results.

		Element Size		
		1 mm size	0.5 mm size	Relative Error
250 N Force	Lateral Contact Area (mm^2)	275.299	281.149	2%
	Medial Contact Area (mm^2)	169.533	172.733	2%
	MAX Contact Pressure (MPa)	1.643	1.347	22%
	Displacement (mm)	-0.279035	-0.278212	0%
	MAX Von Mises (MPa)	0.992	0.879	13%
500 N Force	Lateral Contact Area (mm^2)	330.927	332.567	0%
	Medial Contact Area (mm^2)	204.755	206.784	1%
	MAX Contact Pressure (MPa)	2.287	2.082	10%
	Displacement (mm)	-0.392441	-0.392439	0%
	MAX Von Mises (MPa)	1.475	1.494	1%

3 Ligament Fiber Creation

Ligaments attachment sites were found from a combination of visually identified landmarks during cadaveric dissection and subsequently surface scanned and regions identified based on anatomical descriptions as defined in the main article. Following identification of the site of the ligament, individual fibers were created based on identification of individual end points on each surface. For each ligament site, a 2D ellipse was fitted to the 3D surface of the attachment site, resulting in an ellipse, with a center, and major and minor axis length in a 2D plane defined with a centroid and surface normal. The number of desired fibers was chosen to match existing work from the DU Team of the KneeHub project (SimTK: Reproducibility in Simulation-Based Prediction of Natural Knee Mechanics: Project Home, n.d.; Erdemir et al., 2019; Rooks et al., 2021; Andreassen et al., 2023). Given the desired number of fibers, equidistant points were determined along the major axis of the fitted ellipse and used as the endpoints of individual ligament fibers (Figure 2).

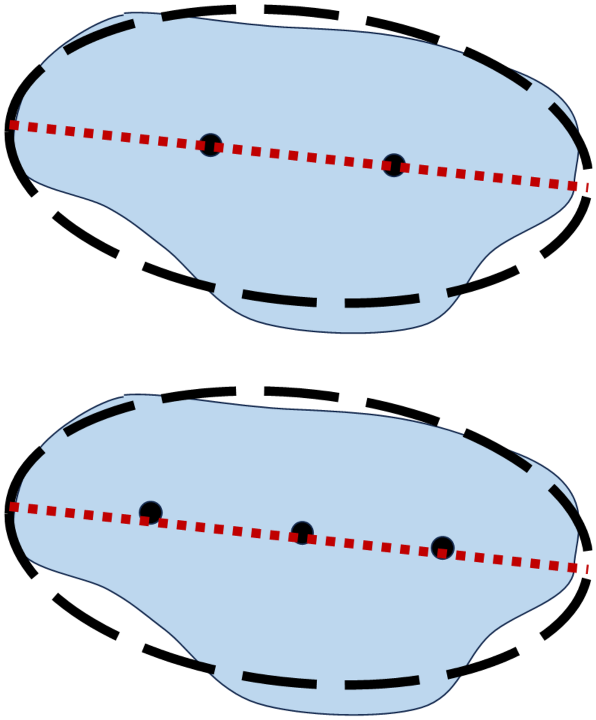

Figure 2: Chosen points for individual fibers for a given attachment region. The blue region is the identified ligament attachment site from the original imaging or literature. The black dashed line is an approximate ellipse fit to this region. The dotted red line is the major axis of the ellipse. The black dots are the chosen ligament attachment sites for the individual fibers. The location of the points is based on the number of desired fibers (Top) 2 fibers or (Bottom) 3 fibers.

4 Cartilage Calibration

As a fully deformable cartilage representation would likely have little effect on the predicted kinematics of the knee (Halloran et al., 2005), an isotropic linear elastic (pressure overclosure) representation was used to decrease simulation run time. However, pressure overclosure representations are sensitive to geometry (Fitzpatrick et al., 2010) and require calibration.

Cartilage material properties were calibrated using a separate set of analyses created in Abaqus/Explicit and Abaqus/Standard for both specimens and all cartilage geometries. Cartilage calibration included only the femoral cartilage and the tibial medial and lateral cartilage. Cartilage was modeled as continuum deformable isotropic linear elastic (Young’s Modulus = 12 MPa and Poisson’s Ratio = 0.45) (Ramaniraka et al., 2005; Fitzpatrick et al., 2010; Trad et al., 2018) in Abaqus/Standard. The bony interface surfaces of all cartilages were rigidly fixed. The articular surfaces were defined with a hard penalty contact relationship and allowed to contact one another without penetration. The deformable cartilage of the femur was then brought into contact with the deformable tibial cartilage by a linear ramping SI load (connector load in Abaqus) from 0 - 500N of compression. The resulting force-displacement curves in the SI direction and contact area characteristics were recorded. A separate set of models was created in Abaqus/Explicit with the same cartilage geometries; however, the cartilage was modeled as rigid-rigid with a tri-linear pressure overclosure relationship (Halloran et al., 2005; Fitzpatrick et al., 2010; Huff et al., 2020).

Calibration of the pressure-overclosure relationship for each model used in Aim 1 and 2 was done relative to its matched Implicit (Abaqus/Standard) model. Using ISight (Dassault Systemes, France) and the methodology described in (Huff et al., 2020), pressure overclosure relationships were calibrated to match the predicted force-displacement curves (Figure 3A) in the SI direction to those previously determined from the continuum Implicit models by minimizing the root-mean-squared error (RMSE) between the predicted displacements at corresponding forces. The resulting pressure overclosure relationships were validated quantitatively by comparing the predicted contact areas at maximum force. Additional validations were done qualitatively by comparing the resulting contact pressure plots. The resulting pressure overclosure relationships were nearly identical for all models for a given specimen (< 1 % different), so the values for the *CTS* models were used hereafter and are shown in Figure 3B and Table 2. The resulting pressure-overclosure relationships were applied to the material definition for the cartilage of the complete knee models for each specimen.

Table 2: Calibrated pressure overclosure relationships for contact between femoral and tibial cartilage for each specimen.

Overclosure (mm)	Specimen 1 Pressure (MPa)	Specimen 2 Pressure (MPa)
-0.01	0.00373	0.00373
0.00	0.00373	0.00746
0.05	0.32448	0.05221
0.10	0.64895	0.26480
0.20	1.33520	1.01445

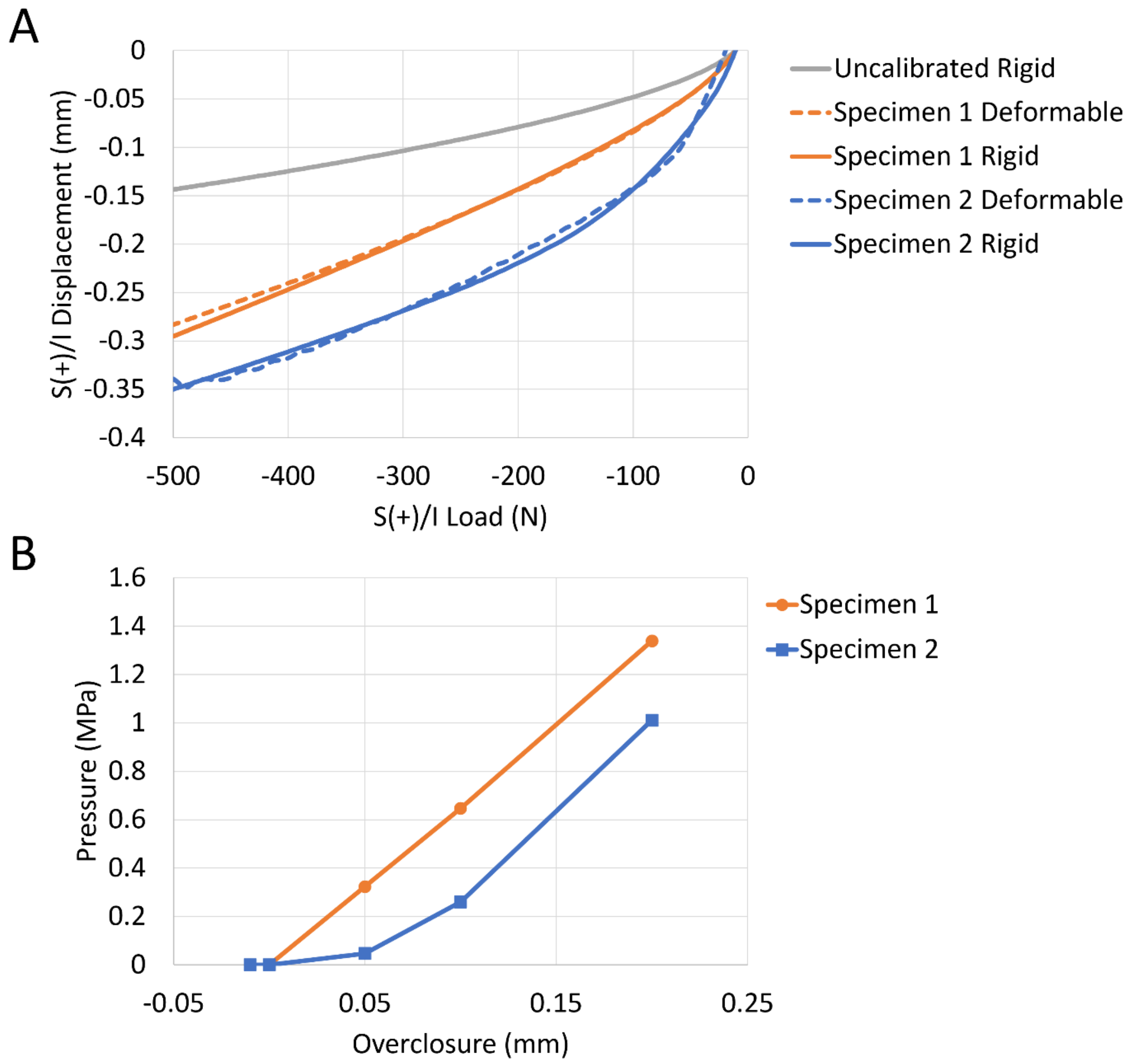

Figure 3: Calibration of rigid-rigid pressure overclosure contact relationships for both specimens. (A) Graphs of compressive force vs contact displacement of femoral cartilage into tibial cartilage. Dashed lines are the results for the specimen geometries deformable models using linear-elastic material model with high Poisson ratio. Solid lines show original uncalibrated rigid-rigid material property and result after material calibration. (B) Resulting pressure overclosure relationships for two specimens following material calibration. Values not in calibrated region are extrapolated using linear relationship based on previous two known values.

5 References

Anderson, A. E., Ellis, B. J., and Weiss, J. A. (2007). Verification, validation and sensitivity studies in computational biomechanics. *Comput Methods Biomech Biomed Engin* 10, 171–184. doi: 10.1080/10255840601160484

Andreassen, T. E., Laz, P. J., Erdemir, A., Besier, T. F., Halloran, J. P., Imhauser, C. W., et al. (2023). Deciphering the "Art" in Modeling and Simulation of the Knee Joint: Assessing Model Calibration Workflows and Outcomes. *J Biomech Eng* 145, 1–13. doi: 10.1115/1.4063627

Erdemir, A., Besier, T. F., Halloran, J. P., Imhauser, C. W., Laz, P. J., Morrison, T. M., et al. (2019). Deciphering the "Art" in Modeling and Simulation of the Knee Joint: Overall Strategy. *J Biomech Eng* 141, 1–10. doi: 10.1115/1.4043346

Fitzpatrick, C. K., Baldwin, M. A., and Rullkoetter, P. J. (2010). Computationally efficient finite element evaluation of natural patellofemoral mechanics. *J Biomech Eng* 132, 1–8. doi: 10.1115/1.4002854

Halloran, J. P., Petrella, A. J., and Rullkoetter, P. J. (2005). Explicit finite element modeling of total knee replacement mechanics. *J Biomech* 38, 323–331. doi: 10.1016/j.jbiomech.2004.02.046

Huff, D. N., Myers, C. A., and Rullkoetter, P. J. (2020). Impact of alignment and kinematic variation on resistive moment and dislocation propensity for THA with lipped and neutral liners. *Biomech Model Mechanobiol* 19, 1297–1307. doi: 10.1007/s10237-020-01359-8

Ramaniraka, N. A., Terrier, A., Theumann, N., and Siegrist, O. (2005). Effects of the posterior cruciate ligament reconstruction on the biomechanics of the knee joint: A finite element analysis. *Clinical Biomechanics* 20, 434–442. doi: 10.1016/j.clinbiomech.2004.11.014

Rooks, N. B., Schneider, M. T. Y., Erdemir, A., Halloran, J. P., Laz, P. J., Shelburne, K. B., et al. (2021). Deciphering the "art" in Modeling and Simulation of the Knee Joint: Variations in Model Development. *J Biomech Eng* 143, 1–12. doi: 10.1115/1.4050028

SimTK: Reproducibility in Simulation-Based Prediction of Natural Knee Mechanics: Project Home (n.d.). Available at: https://simtk.org/projects/kneehub. (Accessed November 30, 2018).

Trad, Z., Barkaoui, A., Chafra, M., and Tavares, J. M. R. S. (2018). *FEM Analysis of the Human Knee Joint*. Cham: Springer International Publishing. doi: 10.1007/978-3-319-74158-1
